# Comparative analysis of machine learning approaches for heatwave event prediction in India

**DOI:** 10.1038/s41598-025-04634-9

**Published:** 2025-07-01

**Authors:** Ritesh Choudary V, Anita Christaline Johnvictor, Prem Sankar N

**Affiliations:** 1 School of Computer Science and Engineering, Vellore Institute of Technology, Chennai Campus, Chennai, India; 2 Centre for Neuro Informatics, Vellore Institute of Technology, Chennai Campus, Chennai, India; 3 Centre for Healthcare Advancement, Innovation and Research, Vellore Institute of Technology, Chennai Campus, Chennai, India

**Keywords:** Machine learning, Classification, Climate change, Heatwaves, Human health, Animal health, Climate sciences, Environmental sciences

## Abstract

Heatwaves, are identified as prolonged durations of unusually high temperatures, which pose significant threats to human health, animal health and agriculture. With the increasing frequency and intensity of heatwaves driven by climate change, accurate and early prediction of these extreme weather events is crucial for effective mitigation and adaptation. This research paper conducts a comparative analysis of various machine learning models for heatwave event classification using a time series dataset from a weather station in the equatorial region of India, specifically Chennai, Tamil Nadu. The study evaluates the performance of models including Random Forest, Convolutional Neural Networks, LightGBM, Long Short-Term Memory Networks, Transformer Networks, Support Vector Machines, Graph Neural Networks, Extreme Gradient Boosting and Autoencoders for Anomaly Detection in heatwave. The challenges posed by class imbalance and the limitations of traditional oversampling techniques are discussed, with insights into effective strategies for improving prediction accuracy. Accurate prediction of heatwaves enables mitigation plans to protect humans, animal and plants.

**Supplementary Information:**

The online version contains supplementary material available at 10.1038/s41598-025-04634-9

## Introduction

Extreme weather events have become a pressing concern in recent years, with heatwaves emerging as one of the most impactful phenomena. Defined by extended durations of excessively high temperatures, heatwaves tend to have severe consequences, including adverse health effects, crop damage, and infrastructure strain^1^. Heatwaves have profound impacts on multiple aspects of life and the environment^2^. Healthwise, they increase the risk of illnesses related to heart and fatalities, particularly among vulnerable people including children, elderly and individuals with pre-existing health conditions^3^. From an agricultural perspective, heatwaves can cause substantial damage to crops, leading to reduced yields and food insecurity^4,5^. Additionally, the strain on infrastructure, particularly energy systems, can result in power outages and increased operational costs. The ecological effects of heatwaves are also significant, disrupting wildlife behaviour, breeding patterns, and overall ecosystem stability^6,7^. Understanding and addressing these impacts are crucial for developing effective mitigation and adaptation strategies.

The increasing frequency and intensity of these events, largely attributed to climate change, emphasize the need for reliable predictive models. Accurate forecasting of heatwaves can facilitate timely interventions and adaptive measures to mitigate their impacts^8^. However, predicting heatwaves presents unique challenges, primarily due to the class imbalance inherent in time series datasets, where heatwave events are infrequent compared to non-heatwave periods. This paper aims to address these challenges by evaluating various machine learning models and exploring methods to handle class imbalance effectively. This research work focuses on the heatwave scenario in India especially in the southern region of Tamil Nadu which is close to equator. The Indian Meteorological Department (IMD) provides a comprehensive definition of heatwaves, which is crucial for understanding the criteria used in heatwave prediction^9^. According to IMD, heatwaves are identified when maximum temperatures reach or exceed 40 °C in plains and 30 °C in hilly regions^10,11^. These thresholds are based on typical temperature norms for different geographical areas. A heatwave is declared when there is a departure from normal temperature ranges between 4.5 and 6.4 °C, while a severe heatwave occurs when this departure exceeds 6.4 °C. Additionally, heatwaves are classified based on actual maximum temperature, where a heatwave is declared if the temperature reaches or surpasses 45 °C, and a severe heatwave if it reaches 47 °C or higher. These criteria must be met in a minimum of two stations within a meteorological sub-division for two successive days to be officially recognized as a heatwave.

Early and accurate predictions of heatwaves play a vital role in mitigating their adverse effects^12^. Timely forecasts enable the implementation of preventive measures, such as public health advisories, establishment of cooling centers, and adjustments to agricultural practices^13^. These measures can significantly reduce the health impacts and economic costs associated with heatwaves^14^. Furthermore, accurate predictions facilitate better preparedness and response, allowing governments and organizations to allocate resources more efficiently and implement contingency plans^15^. In the long term, understanding heatwave patterns and improving prediction accuracy contribute to developing robust adaptation strategies, enhancing societal resilience against future extreme weather events^16^.

Predicting heatwaves using time series weather data presents several challenges, primarily due to high class imbalance^17,18^. Heatwaves are rare events compared to the prevalence of non-heatwave periods, resulting in a significant imbalance in the dataset^19–21^. This imbalance complicates the modelling process, as traditional techniques for handling class imbalance, such as Synthetic Minority Over-sampling Technique (SMOTE) and Adaptive Synthetic (ADASYN), are not applicable to time series data^22–25^. These methods can disrupt the temporal dependencies inherent in time series, making them unsuitable for predicting events based on sequential data. To address these challenges, this study explores alternative machine learning techniques, including graph-based models and deep learning approaches, to improve heatwave detection accuracy without compromising temporal dependencies. By filling the existing research gap in time series-based heatwave forecasting, this study aims to enhance predictive capabilities and contribute to improved disaster preparedness and climate resilience^26–29^. The prime objectives of this research work are

- *The Need for Accurate Heatwave Prediction in Tropical Regions* Tamil Nadu has experienced an increase in heatwave frequency, yet most forecasting models are built on datasets from temperate or continental climates. This study aims to develop models specifically trained on local climatic conditions to improve regional prediction accuracy.
- *Machine Learning Model Selection and Optimization* The study sought to determine which machine learning models perform best in heatwave classification, comparing traditional methods (Random Forest, SVM, XGBoost) with advanced deep learning architectures (CNNs, LSTMs, Transformers, GNNs, and Autoencoders). The objective was to identify the most effective approach for balancing accuracy, recall, and computational efficiency.
- *Addressing Class Imbalance without compromising temporal structure* Many prior studies struggle with class imbalance in extreme weather event prediction. Instead of using synthetic oversampling (which disrupts time-series continuity), this study investigates alternative techniques such as class weighting and anomaly detection. The goal was to develop a methodology that enhances rare event detection while preserving sequential dependencies in weather data.

## Existing literature

Existing literature shows several research works carried out in different parts of the world to understand and estimate the heat waves in their local regions^24,30–32^. Different metrics have been used by researchers to identify and classify heat waves^33^. While many of the researchers focus on heat wave predictions, others have equally contributed to studies related to health issues due to excess heat waves.

Research by Jacques et al.^34^ has been implemented with Convolutional Neural Network (CNN), large-class under-sampling, transfer learning. These strategies enhance the model’s accuracy in predicting extreme events, even when they are underrepresented in the dataset. They claim that the benefits of Large Training Datasets in deep learning model’s success is in part due to having access to simulated climate data for a duration of 1000 years. This large dataset enables the model to effectively identify patterns and anomalies related to extreme heatwaves.

Work by Li et al.^26^, uses Attention-Based Graph Neural Network and is based on the effectiveness of GNN in Heatwave prediction. Their proposed Graph Neural Network (GNN) model proves to be highly effective, achieving 94.1% accuracy in predicting regional heatwaves. This result is based on daily weather data from 91 ground stations across the Contiguous United States (CONUS). The Spatiotemporal Patterns and Climate Dynamics claimed by them is that the GNN model accurately captures and replicates spatiotemporal patterns in climate dynamics, enhancing the understanding of heatwave occurrences. Their model Performance on Heatwave Prediction has an overall Accuracy of 94.1% on the validation dataset, Recall of 58.5% and Precision of 62.5%. The model can be adjusted to Trade-offs to prioritize higher recall (sensitivity) or precision according to practical requirements. Prediction Lead Time reported is, the performance declines with longer lead times (Cout), making it suitable for immediate warnings but more challenging for long-term predictions.

Polynomial Fitting Model, Geographically Weighted Regression (GWR) Model and Heat Wave Risk Model have been implemented by Mengxi Liu et al.^35^. Their GWR model outperforms the Kriging model in generating spatially continuous temperature maps. They report Mean Absolute Error (MAE) comparison with the GWR model that demonstrates a lower MAE (0.7898) compared to the traditional Kriging model (0.8415). Absolute Value Error and Variance for Polynomial Fitting have been evaluated across various land types, the errors ranged between 0.5 and 0.8 K, with a variance of 0.4 K. Their accuracy meets the requirements for assessing heat wave conditions.

In their work titled HeatwaveR package in R for detecting heatwaves using daily diurnal temperature range (DTR). CMIP5 model for future heatwave projections, Kapwata et al.^36^ define heatwave as exceeding a DTR threshold of 12.8 °C for 2 or more consecutive days. Temporal Coverage includes past data from 2014 to 2019 and future projections from 2020 to 2039. Their Spatial Coverage analyzes 50 out of 52 district municipalities in South Africa for scenarios considered with RCP 4.5, which is moderate emissions and high emissions with RCP 8.5. Their metrics include duration, frequency and intensity of heatwaves.

Research by Mehiriz^37^, include data gathered on oral information through phone in two different years in the city of Longueuil, Canada. This data is a sample of individuals who are more vulnerable to heatwaves. The perceived control did not moderate the relationship between attitudes and norms on intentions. Additionally, older individuals were found to be less likely to adopt heat protection actions compared to other age groups, and chronic health conditions did not significantly increase the likelihood of adoption.

Work by Maharana et al.^38^, use the CORDEX-CORE dataset, which includes high-resolution climate model simulations for the South Asian region. The model used employed the RegCM4 regional climate model, driven by sea surface temperatures, lateral boundary conditions from three different CMIP5 GCM models. Their model’s performance was evaluated against ERA5 reanalysis data to assess its ability to simulate historical climatological temperature patterns. Their study forecasts a substantial rise in both the frequency and intensity of heatwaves across India by the end of the twenty-first century under both the RCP2.6 and RCP8.5 scenarios. Their findings highlight the necessity of implementing effective mitigation strategies, such as reducing greenhouse gas emissions and enhancing adaptive capacities, to alleviate the negative impacts of extreme heat events on the population.

The study by Welch et al.^39^, utilized boosted regression tree models (BRTs) to model the effects of four North Pacific marine heatwaves on the spatial distributions of 14 marine top predator species. Their study observed significant variability in responses among different species and marine heatwaves (MHWs). Some predators faced nearly complete habitat loss and range compression, while others saw habitat increases and range expansion. The severity of impacts on species was generally greater in areas with the highest MHW temperature anomalies, with coastal and southern species particularly affected by warmer temperatures during specific MHW events. The study emphasizes the necessity for dynamic and adaptable management strategies to effectively address the impacts of MHWs on marine top predators.

Research by Yang et al.^40^, adopt various methods for Detection of Extreme Heatwaves: Daily maximum temperature data for the warmer season (May–September) from 1979 to 2021 over the Contiguous United States (CONUS) were obtained Hourly Air Temperature Data: Hourly air temperature data for 520 urban weather stations across the CONUS were retrieved covering the same period (1998–2021) as the extreme heatwave analysis. The performance metric for each of these methods were intensity, duration, and spatial coverage. Their inferences include, identification of significant heatwave events in the CONUS based on their intensity, duration, and spatial extentThe strength of causality and population metrics show positive correlation, indicating that human activities significantly impact the causal interactions among cities during heatwaves.

Wu et al.^41^, utilized daily maximum and minimum temperature data from the ERA5 reanalysis dataset (1979–2020). They identified heatwave events according to 90th percentile for a 15 day moving window and classified heatwaves into independent daytime, independent nighttime, and compound types. Their metrics include accuracy in categorizing heatwave events into daytime, nighttime, and compound types. Their inference includes, successful identification and classification of three distinct types of heatwaves worldwide. Investigation of the unique physical mechanisms for each type by composite analysis, contribution to understanding the drivers of various heatwave types, which can inform future research and adaptation strategies.

Research by Han et al.^42^, utilized tide gauge data, satellite altimeter data, and ocean reanalysis products. They used the climate Mode Indices: Calculated various indices including the nino 3.4 index for ENSO, the dipole mode index for the Indian Ocean Dipole (IOD), and wind shear index for Indian monsoon variability. Their inferences are

- *Detection of Extreme Events* The study identified extremes in height and extremes in the compound height-heat along the Indonesian coast of the Indian Ocean through observations and model simulations.
- *Anthropogenic Influence* global sea level rise and decadal changes were found to play a role in the increased presence and intensity of HEXs during specific periods, particularly from 2010 to 2017.Climate Variability: ENSO and IOD were identified as significant drivers of climate variability impacting sea level extremes in the region.
- *Model Consistency* The study emphasized the alignment between various datasets and model simulations in detecting and simulating extreme events, which enhances confidence in the results.
- *Implications* The findings highlighted the necessity of understanding the interaction between anthropogenic warming and climate variability in shaping regional extremes, with implications for social, environmental, and ecological stresses in the area.

Park et al.^43^ have used Random Forest Regression owing to its ability to handle large and diverse datasets. The Boruta algorithm was used for variable selection, confirming the importance of all collected variables. Their metric for evaluation is Mean Absolute Error, Root Mean Squared Logarithmic Error, Root Mean Squared Error and Coefficient of Determination (R^2^). This model outperformed traditional regression models, achieving an R^2^ value of 0.804. The study suggests that this model could be applied for proactive disaster response decision-making to reduce heatwave-related losses.

Analysis of historical temperature data (1958–2011), Calculation of Excess Heat Factor (EHF), Climatological distribution analysis and Pilot national heatwave forecasting service (2013/2014 summer) were conducted by Nairn et al.^33^. They infer that the EHF is a valuable tool for identifying and predicting heatwaves in Australia. There has been a notable increase in both the intensity and frequency of heatwaves over time. The impacts of heatwaves differ by region, with southern Australia experiencing more severe heatwaves. The EHF methodology can be utilized to enhance heatwave forecasting and adaptation strategies.

Research by Albergel et al.^44^ and Gustin et al.^45^ have studied the internal air temperature characteristics during heatwaves. Although both ARX and ARMAX models were used and compared, ARX models provided simpler and more consistent predictions. The perfomace analysis is based on

- *Accuracy Metrics* Mean Bias Error (MBE), Root Mean Square Error (RMSE), Mean Absolute Error (MAE) and adjusted coefficient of determination (R^2^_adj).
- *Reliability Assessment* 95% prediction intervals to indicate the reliability of forecasts.
- *Comparison of ARX and ARMAX Models* Evaluation based on predictive accuracy metrics across different forecasting horizons.

Weirich et al.^46^, have utilized ensemble forecast and hindcast data from ECMWF System 5 (SEAS5) spanning from 1981 to 2016. Their study shows that SEAS5 has predictive skill for local heatwave indices across Europe, particularly in the eastern and southern regions. However, the model’s reliability in forecasting the location and timing of severe heatwaves remains relatively low. The research indicates that heatwave seasonal prediction could be enhanced through sensitivity experiments and the creation of statistical prediction systems focused on heatwave indices. Overall, seasonal forecasts provide valuable insights into the likelihood and characteristics of heatwaves, supporting societal adaptation and mitigation efforts.

Research by Jacox et al.^47^ utilized an ensemble of global forecasts. They infer that Marine heatwaves (MHWs) can be predicted with considerable skill up to 12 months ahead, with variation depending on the region and season. Forecasting skill is strongest in the tropics and the extratropical Pacific, while it is lower in the South and along the West Currents. ENSO improves the forecast skill for marine heatwaves (MHWs), particularly in the Indian and East Pacific. Accurately predicting the onset, intensity, and duration of MHWs facilitates proactive decision-making and enhances resilience in marine ecosystems.

Based on the above review of the literature, we intend to implement this research with the following key novelties:

- *Comparative Evaluation of a Wide Range of Machine Learning Models* Unlike previous studies that focus on single models or narrow model comparisons, this study evaluates a broad spectrum of nine machine learning approaches, including CNNs, Transformers, Graph Neural Networks, Autoencoders, and traditional ensemble methods (XGBoost, LightGBM, Random Forest, etc.). This extensive comparison provides a benchmark for heatwave prediction models, highlighting the strengths and weaknesses of different approaches under real-world conditions.
- *Handling Class Imbalance in Time-Series Heatwave Prediction* Heatwaves are rare events, leading to severe class imbalance in training data. Many past studies rely on oversampling techniques (e.g., SMOTE), which disrupt time-series dependencies. This study avoids SMOTE and instead leverages class weighting, anomaly detection, and deep learning-based augmentation, suggesting better ways to improve model sensitivity to heatwave events.
- *Integration of Graph-Based and Deep Learning Methods* This research explores Graph Neural Networks (GNNs) for heatwave prediction, which is rare in existing literature. While GNNs were found to be less effective for this specific task, the findings contribute valuable insights into the limitations and potential applications of graph-based models in meteorology. The study also compares Transformer-based architectures, which are relatively new in climate forecasting.

## Methodology and implementation

**Fig. 1:**
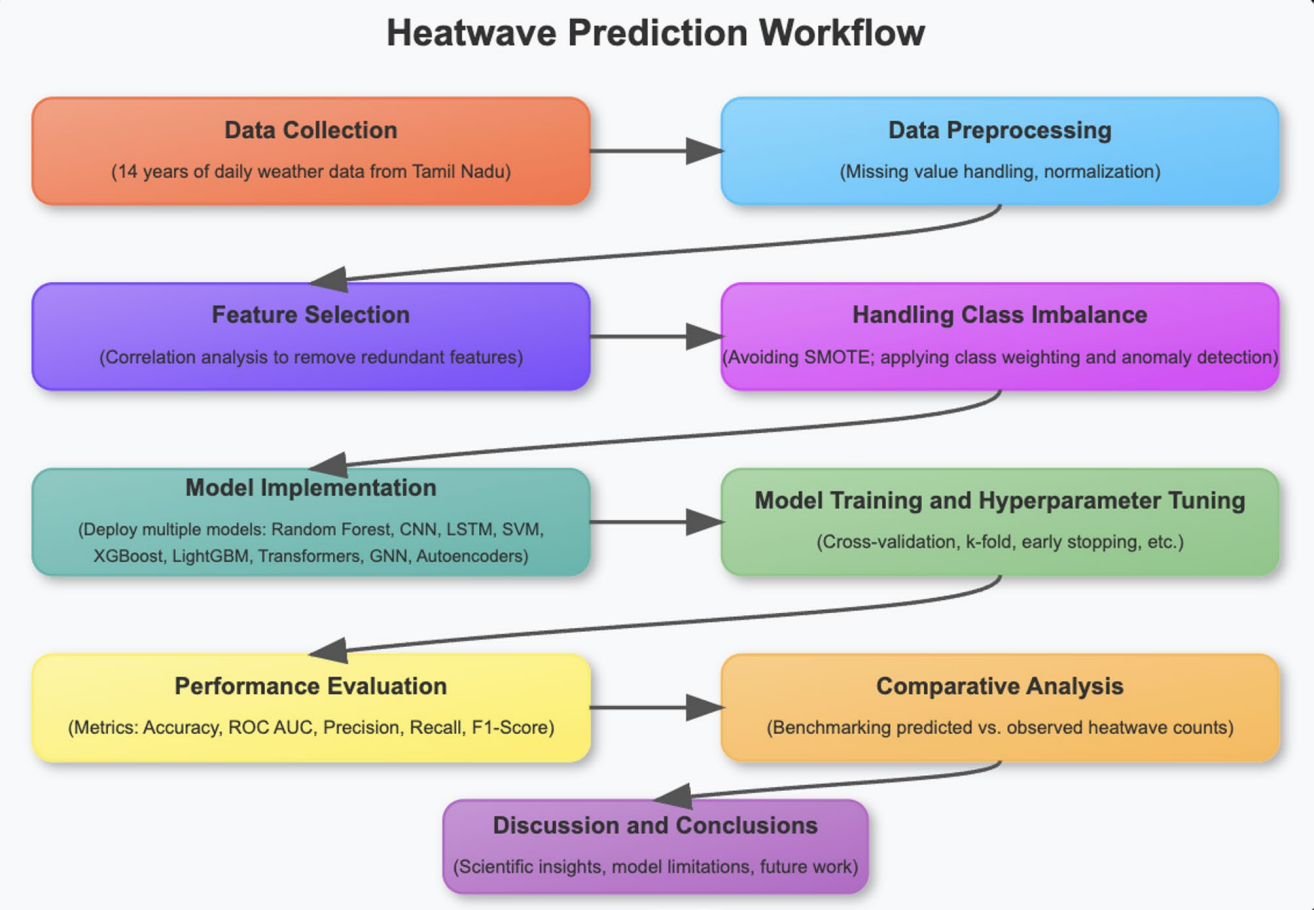
Heat prediction workflow implemented in this research.

This research work implements nine different machine learning approaches for predicting heatwaves. These methods include, Random Forest, Convolutional Neural Networks (CNN), LightGBM, Long Short-Term Memory Networks (LSTM), Transformer Networks, Support Vector Machines (SVM), Graph Neural Networks (GNN), Extreme Gradient Boosting (XGBoost) and Autoencoders for Anomaly Detection. Their performance metrics like precision, recall, accuracy, ROC curve and F1 score serve as a tool for predicting the heatwaves. The parameter settings for each of the chosen models include, Data Splitting & Cross-Validation: The dataset was split into 80% training and 20% testing. fivefold cross-validation was applied to ensure robustness, particularly for models sensitive to class imbalance. Stratified sampling was used to maintain the distribution of heatwave versus non-heatwave instances. The hyperparameters are as shown in Table 1. The workflow involved in this research implementation is shown in Fig. 1.

**Table 1:** Key hyperparameters settings.

Model	Key hyperparameters	Optimization details
Random forest	n_estimators = 100, max_depth = 10, class_weight = ‘balanced’	Grid search optimization
XGBoost	learning_rate = 0.1, n_estimators = 200, max_depth = 6, subsample = 0.8	Early stopping used
LightGBM	learning_rate = 0.1, num_leaves = 31, n_estimators = 150	Grid search optimization
Support vector machine (SVM)	kernel = ‘linear’, class_weight = ‘balanced’, C = 1.0	Default hyperparameters
Convolutional neural network (CNN)	filters = 64, kernel_size = 2, dropout = 0.5, dense_units = 50	Adam optimizer, learning rate 0.001
LSTM	units = 50, dropout = 0.5, batch_size = 32, epochs = 50	Adam optimizer, learning rate 0.001
Transformer	num_heads = 4, ff_dim = 32, dropout = 0.1	Class weighting used
Graph neural network (GNN)	hidden_dim = 16, learning_rate = 0.001, num_epochs = 50	Adam optimizer
Autoencoder	encoding_dim = 14, loss = ‘mse’, epochs = 50	Feature extraction used

### Dataset

**Fig. 2:**
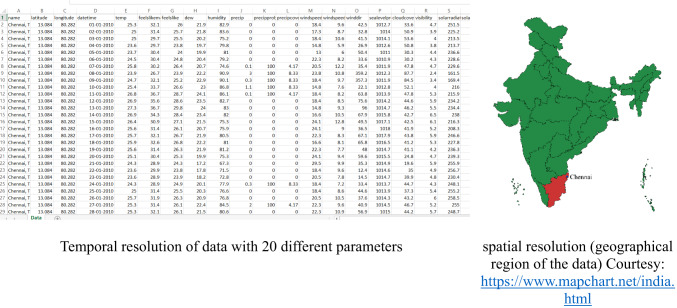
Temporal and spatial resolution of the dataset with 20 different parameters from Tambaram, Chennai, India.

**Fig. 3:**
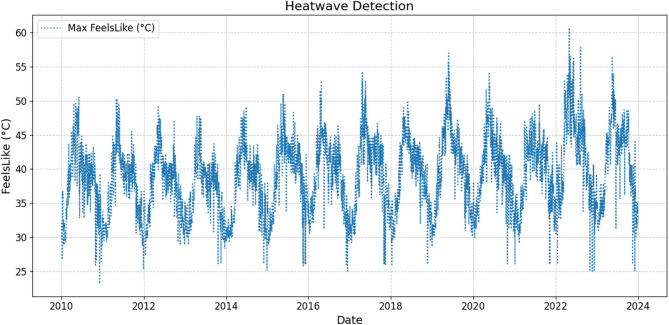
Temperature distribution over the 14 years of the dataset.

**Fig. 4:**
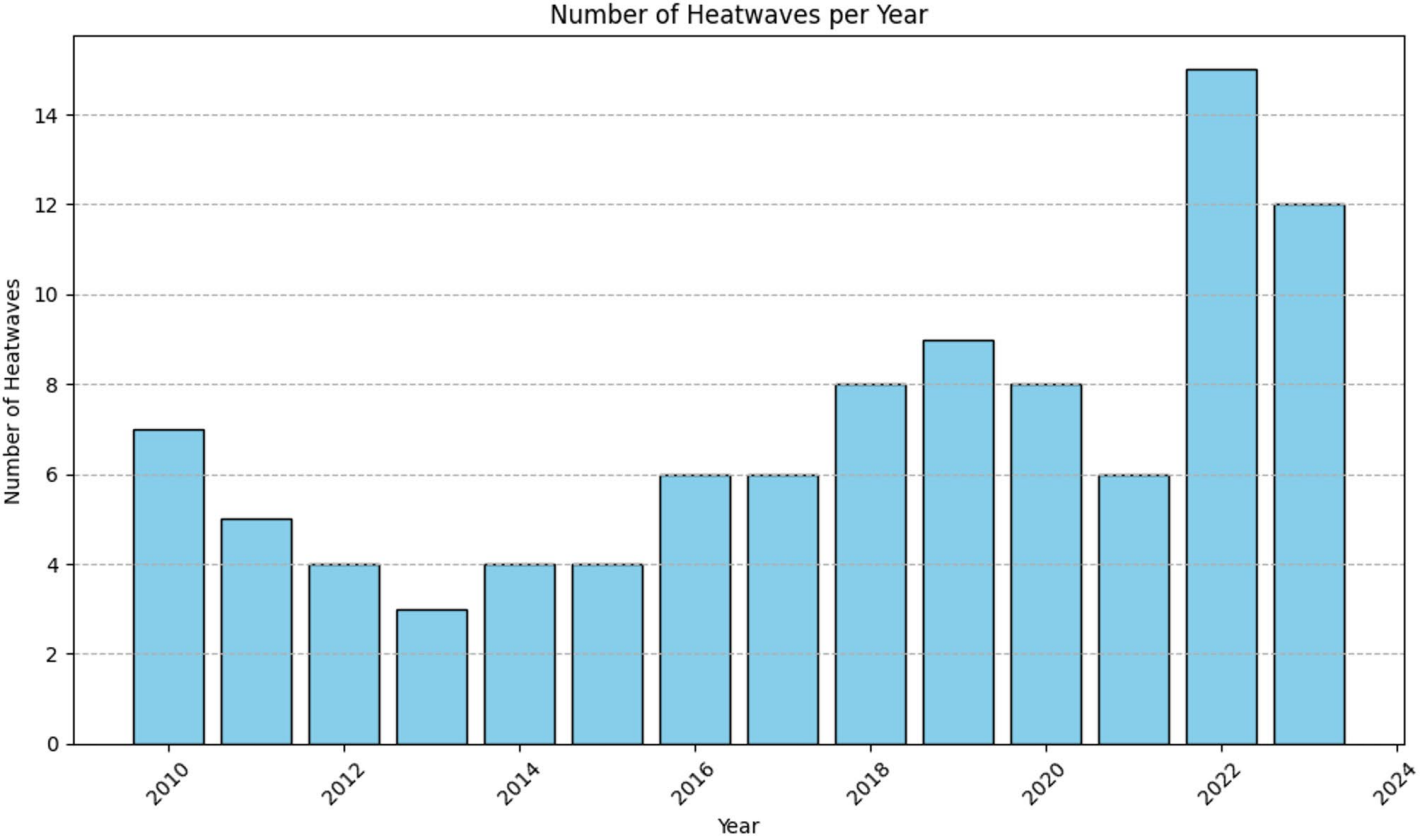
The number of heat waves in each year.

**Fig. 5:**
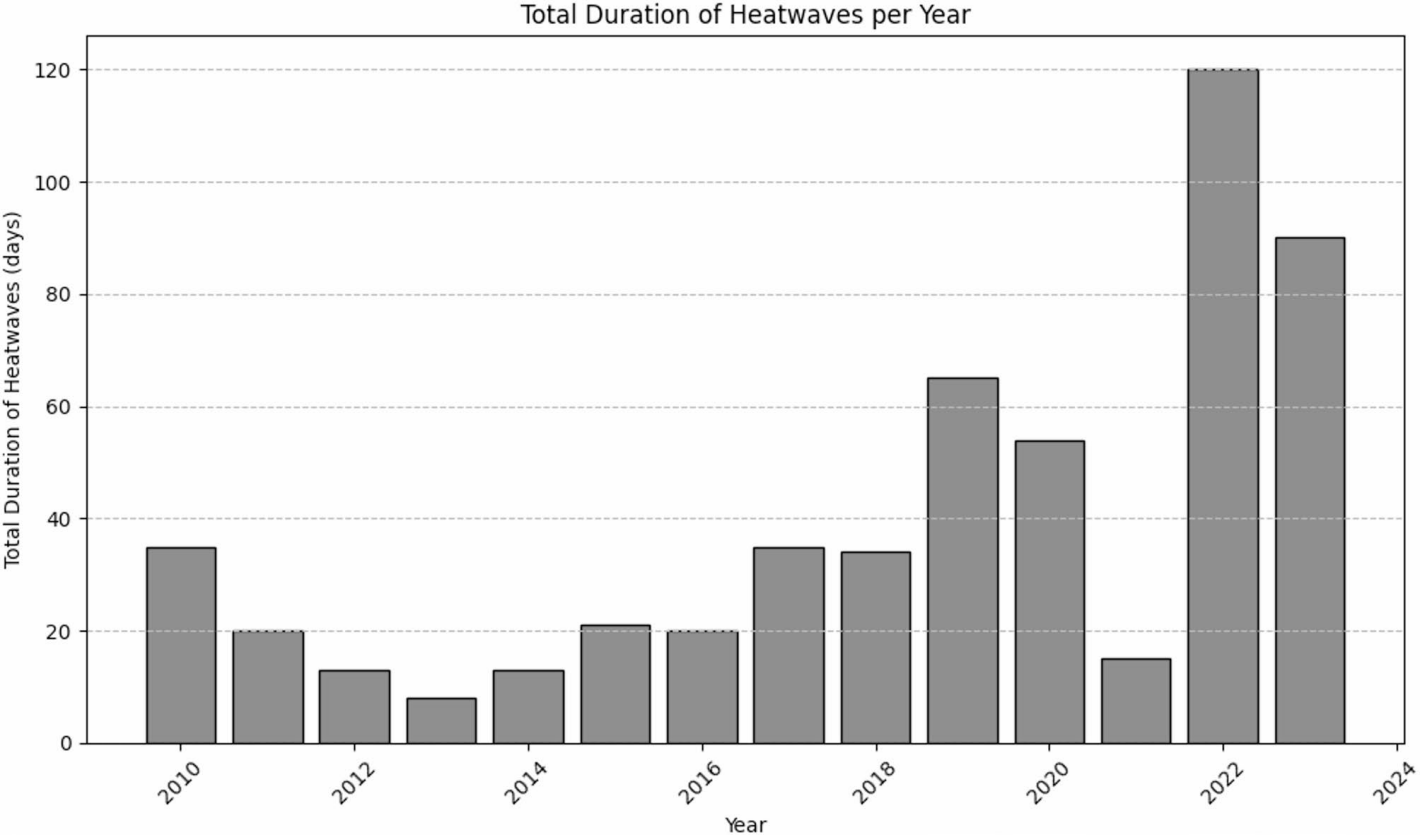
Duration of the heatwaves for consecutive days.

This study utilizes a comprehensive dataset encompassing 14 years of weather observations recorded at a weather station in Tambaram, Chennai, Tamil Nadu, India^48^. The source of the data collected is from https://www.visualcrossing.com/weather/weather-data-services. There are 20 different parameters. The temporal resolution is 13 years from 2010 to 2023. The spatial resolution includes the geographical region of Tambaram in Chennai, India. These are shown in Fig. 2. The Latitude, Longitude, DateTime, Temperature, feelslikemax, feelslikemin, dew, humidity, precipitation, precipitation probability, precipitation cover, windspeed, windspeedmean, wind direction, sea level pressure, cloud cover, visibility, solar radiation, solar energy, UV index. To ensure data consistency, missing values were handled through interpolation, and highly correlated features were removed to prevent redundancy. Feature selection was performed based on correlation analysis to retain only the most relevant predictors for heatwave classification. To avoid data leakage and misleading model performances, feature selection was performed on the training set. The maximum temperature levels over these years has been plotted as in Fig. 3. The number of heat waves in each year is also seen to be variable in the plot in Figs. 4 and 5 shows the duration of the heatwaves in days. In Chennai, the warmest months are May, June, July^49^. While many researchers have studied and analyzed heatwaves over India^50–54^, heatwave prediction over Chennai, Tamil Nadu using machine learning classifiers has not be conducted till now. Our models were tested on data during these months.

The dataset comprises many various meteorological features (out of the total 20 features) essential for weather prediction and analysis, as detailed below:

- *Date-time* The timestamp for each recorded data entry.
- *Temperature Metrics* Air temperature, perceived warmth, and maximum apparent temperature.
- *Humidity and Dew Point* Relative humidity and dew point temperature.
- *Precipitation* Amount of precipitation, probability of precipitation, and fraction of the sky covered by precipitation.
- *Wind Metrics* Wind speed, mean wind speed, and wind direction.
- *Atmospheric Conditions* Atmospheric pressure at sea level, cloud cover, and visibility.
- *Solar Metrics* Solar radiation intensity, solar energy received, and ultraviolet index.

To enhance the efficacy of heatwave classification, feature selection was conducted to isolate the most pertinent predictors. In this study, feature selection was applied to improve model performance by removing redundant and less informative features from the dataset created using a sliding window approach. The method used for evaluating feature relationships was Pearson correlation, which measures the linear correlation between pairs of variables. The Pearson coefficient ranges from − 1 (perfect negative correlation) to + 1 (perfect positive correlation), with 0 indicating no linear relationship. To preserve temporal dependencies, a sliding window of size 7 days was applied to the dataset. For each time window, all feature values from each day in the 7 day sequence were stacked into a single feature vector. This results in features labelled as: TimeStep_0_Feature_0, TimeStep_0_Feature_1, …, TimeStep_6_Feature_n, where: TimeStep_X refers to the day index within the 7-day window (from 0 = oldest day to 6 = most recent day), Feature_Y refers to the original feature index (e.g., temperature, humidity, etc.) as ordered in the dataset before transformation.

Correlation Thresholding

After reshaping, the correlation matrix was computed using Pearson correlation for all pairs of features. The selection logic involved the following criteria:

- Features with an average absolute correlation (mean_abs_corr) above 0.9 were considered too highly correlated and hence redundant.
- Features with a mean absolute correlation below 0.3 were deemed weakly correlated and likely less informative.
- From groups of strongly correlated features, only the most representative feature (the one with the highest correlation to others) was retained.

These filtering steps aimed to:

- Remove multicollinearity, which can degrade model performance,
- Reduce dimensionality, making the learning process more efficient,
- Improve generalization, especially important in imbalanced classification tasks.

After this correlation-based pruning a subset of features was selected using RandomForestClassifier with SelectFromModel, where features contributing less than the mean importance threshold were removed. This combined approach ensured both statistical relevance (via correlation) and predictive relevance (via feature importance).

The final list of selected original features contributing significantly to model accuracy included the following. These represent core meteorological variables known to be associated with heatwave conditions.

Feature_0 = temp

Feature_1 = feelslikemax

Feature_2 = feelslike

Feature_3 = dew

Feature_4 = humidity

Feature_5 = precip

Feature_6 = precipprob

Feature_7 = precipcover

Feature_8 = windspeed

Feature_9 = windspeedmean

Feature_10 = winddir

Feature_11 = sealevelpressure

Feature_12 = cloudcover

Feature_13 = visibility

Feature_14 = solarradiation

Feature_15 = solarenergy

Feature_16 = uvindex

**Fig. 6:**
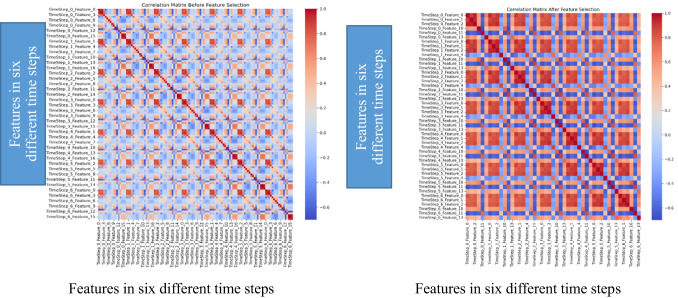
Correlation matrix of features. (**a**) is Correlation matrix before feature selection. (**b**) is Correlation matrix after feature selection.

Features with correlation values below 0.3 or exceeding 0.9 were excluded to mitigate redundancy and multicollinearity. After correlation, few features have been elimated and the final set of features retained for model training includes cloud cover, perceived warmth, maximum apparent temperature, relative humidity, precipitation coverage, sea-level pressure, wind speed, and mean wind speed. Figure 6 illustrates the correlation matrix before and after feature selection, highlighting the reduction in redundancy and multicollinearity among the retained features. As indicated in the colour scale in Fig. 6, it can be noticed that in Fig. 6a, the correlation coefficients are less (blue colour), whereas in Fig. 6b, the correlation coefficients are high (red colour).

### Handling class imbalance in the time series data

Addressing class imbalance in the time series data requires alternative approaches. Traditional oversampling methods like SMOTE are not suitable due to their disruption of temporal dependencies. Instead, the following strategies are recommended:

- *Resampling Strategies* Under sampling the majority class can balance the dataset while preserving the temporal structure. Alternatively, sliding window techniques can generate smaller, balanced sequences from the time series data.
- *Class Weighting* Adjusting model loss functions to emphasize the minority class can improve prediction performance for heatwave events.
- *Data Augmentation* Techniques such as time warping, noise injection, and window slicing can enhance minority class representation without disrupting temporal sequences. Time Series Generative Adversarial Networks (GANs) may also be employed to generate synthetic sequences that maintain temporal coherence.
- *Anomaly Detection* Reframing heatwave prediction as an anomaly detection problem can leverage models adapted for time series data, focusing on identifying rare events within the sequence.

In this implementation, class imbalance in the dataset was handled using class weighting instead of SMOTE, as SMOTE disrupts the temporal dependencies in time-series data. The Random Forest, SVM, and Transformer models utilized built-in class_weight = ‘balanced’, which adjusts the loss function to give higher importance to the minority class (heatwave events), thereby improving recall without excessive false positives. Deep learning models such as CNNs and LSTMs were trained with weighted loss functions, ensuring that rare heatwave occurrences contributed more to the optimization process. Additionally, cross-validation techniques such as k-fold validation were used to prevent overfitting while maintaining robust performance across different data splits. This approach allowed the models to better identify heatwaves while minimizing bias towards the majority class. These techniques were applied on chosen training set to avoid spurious high model skills.

### Performance results of the models

To address the challenge of heatwave prediction, several machine learning models were evaluated. The performance of these models varied, highlighting their strengths and limitations, the performance result figures in terms of confusion matrix, ROC curve, training and Validation loss (for few models) are given as supplementary information in Appendix.

Random Forest:

- *Performance* Random Forest (RF) shows good performance, with a precision of 0.80, recall of 0.67, and F1-score of 0.73. Its test ROC AUC of 0.9956 and accuracy of 0.9912 reflect its strength in distinguishing heatwave from non-heatwave periods. RF, is an ensemble learning method that leverages multiple number of decision trees, each trained on different subsets of the data. This method reduces over fitting and increases generalization. The random sub-sampling of both features and data in each tree mitigates noise and class imbalance issues to some extent. Random forest has been chosen owing to its ability to handle missing data and robustness to overfitting.

Convolutional Neural Networks (CNN):

- *Performance* CNN had strong results, with a precision of 0.73, recall of 0.61, F1-score of 0.67, and a test ROC AUC of 0.9943, test accuracy of 0.9892. It captured complex patterns in the data quite effectively. CNNs are known for excelling in image processing and pattern recognition. Working with time series data, CNNs extract spatial patterns and relationships between meteorological features. CNN are effective in recognizing non linear relationships in data. Also they are adaptable for complex data structures.

Long Short-Term Memory Networks (LSTM):

- *Performance* LSTM struggled in this task, with a precision, F1-score and recall of 0.00. Even though its test ROC AUC was 0.9607 and accuracy was 0.9824. It failed to predict heatwaves. LSTM models are designed for sequential data like time series, but the high class imbalance (few heatwave events compared to non-heatwave periods) makes it difficult for the model to learn meaningful patterns for the minority class. LSTM focuses on dominant patterns in the data and , heavily favors non-heatwave periods. LSTM is implemented in this research as are effective in modeling time series data. Also they have better memory retention of historical data.

Support Vector Machines (SVM):

- *Performance* SVM had a precision of 0.12, recall of 1.00, and F1-score of 0.22. The model had a strong recall but at the cost of precision, meaning it was able to identify all heatwave events but with many false positives., The implemented SVM model prioritized recall, leading to high sensitivity but poor specificity. SVMs is implemented due to its effective high dimensional spaces and clear class separation capability. Also it is less prone to overfitting.

Extreme Gradient Boosting (XGBoost):

- *Performance* XGBoost performed well with a precision of 0.75, recall of 0.50, F1-score of 0.60, and a test ROC AUC of 0.9932. Its performance was slightly below Random Forest but still strong overall. XGBoost is implemented as it works well in structured data by minimizing loss iteratively. It handles imbalanced data better by adjusting weights or by tuning hyperparameters like the learning rate, max depth, and the number of estimators. XCBoost gives better control over overfitting, capable of handling large datasets and missing values.

LightGBM:

- *Performance* LightGBM showed similar performance to XGBoost, with precision, recall of 0.75 and 0.50, F1-score of 0.60, and test ROC AUC of 0.9931. LightGBM is a gradient boosting framework similar to XGBoost but optimized for speed and memory efficiency, which makes it scalable to large datasets and quick to train. Usage of LightGBM supports categorical features and can handle sparse data efficiently. It is also highly efficient on large datasets.

Transformer Networks:

- *Performance* Transformers achieved results similar to XGBoost and LightGBM, with precision and recall values of 0.75 and 0.50, F1-score of 0.60, and test ROC AUC of 0.9932. Transformer networks have attention mechanism which focus on important parts of the input data.

Graph Neural Networks (GNN):

- *Performance* GNN had poor results, with a precision and recall of 0.00, and an F1-score of 0.00. It had a low-test ROC AUC of 0.4714, indicating its ineffectiveness in the heatwave prediction task. The weather dataset didn’t have an inherent graph structure, making GNNs less suitable for this task.

Autoencoders for Anomaly Detection:

- *Performance* The autoencoder achieved a precision of 0.07 and recall of 0.94, with an F1-score of 0.13. It had a test ROC AUC of 0.8768, showing potential for anomaly detection but limited classification ability. Autoencoders are implemented as they give better results for unsupervised anomaly detection tasks. Since heatwaves are rare, the model could identify them as anomalies, but it lacked the accuracy required for classification. This method is preferred as it is effective for detecting outliers and hence reduce dimensionality.

Performance & Error Metrics: Models were evaluated using ROC-AUC, Accuracy, Precision, Recall, and F1-score. Deep learning models (CNN, LSTM, Transformers) used binary cross-entropy loss and Adam optimizer. Autoencoders used mean squared error (MSE) loss for anomaly detection. The best-performing models (CNN, XGBoost, and LightGBM) had ROC-AUC > 0.99, while LSTM and GNN struggled due to class imbalance and unsuitable data structures.

All the performance metrics for the implemented classifiers have been tabulated in Table 2.

**Table 2:** Performance metrics.

Model	Precision	Recall	F1-score	ROC AUC	Accuracy
Random forest	0.70	0.47	0.63	0.9856	0.9812
Convolutional neural network	0.73	0.61	0.67	0.9943	**0.9892**
Long short-term memory	0.00	0.00	0.00	0.9607	0.9806
Support vector machine	0.12	1.00	0.22	0.9511	0.8727
Extreme gradient boosting	0.75	0.50	0.60	0.9932	0.9882
LightGBM	0.75	0.50	0.60	0.9931	0.9863
Transformer networks	0.75	0.50	0.60	0.9932	0.9806
Graph neural network	0.00	0.00	0.00	0.4714	0.4923
Autoencoders for anomaly detection	0.07	0.94	0.13	0.8768	0.8554

Bold value shows the highest value among all.

**Fig. 7:**
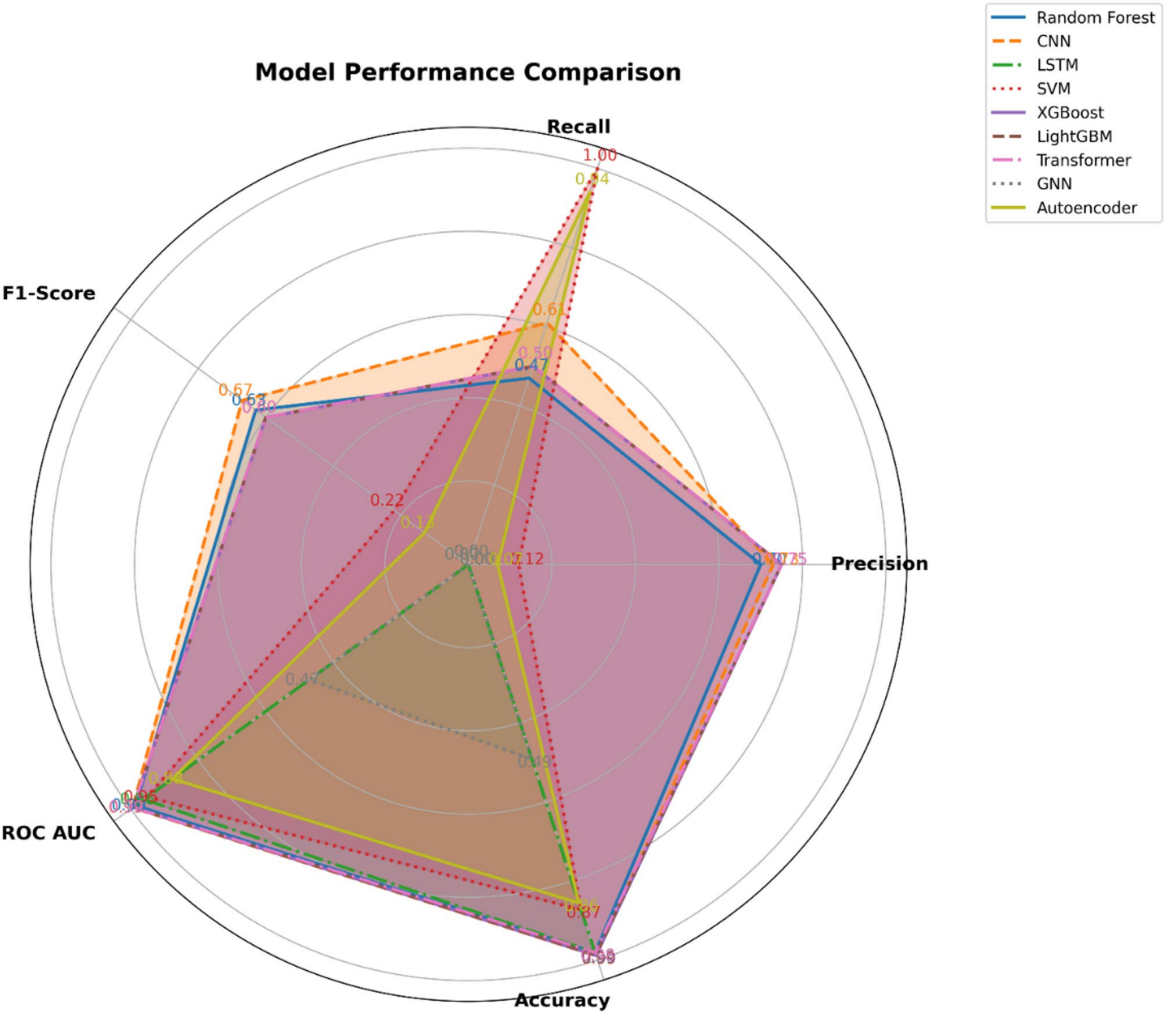
Radar chart for overall performance.

The overall performance of all the classifiers has been shown as Radar chart in Fig. 7.

**Fig. 8:**
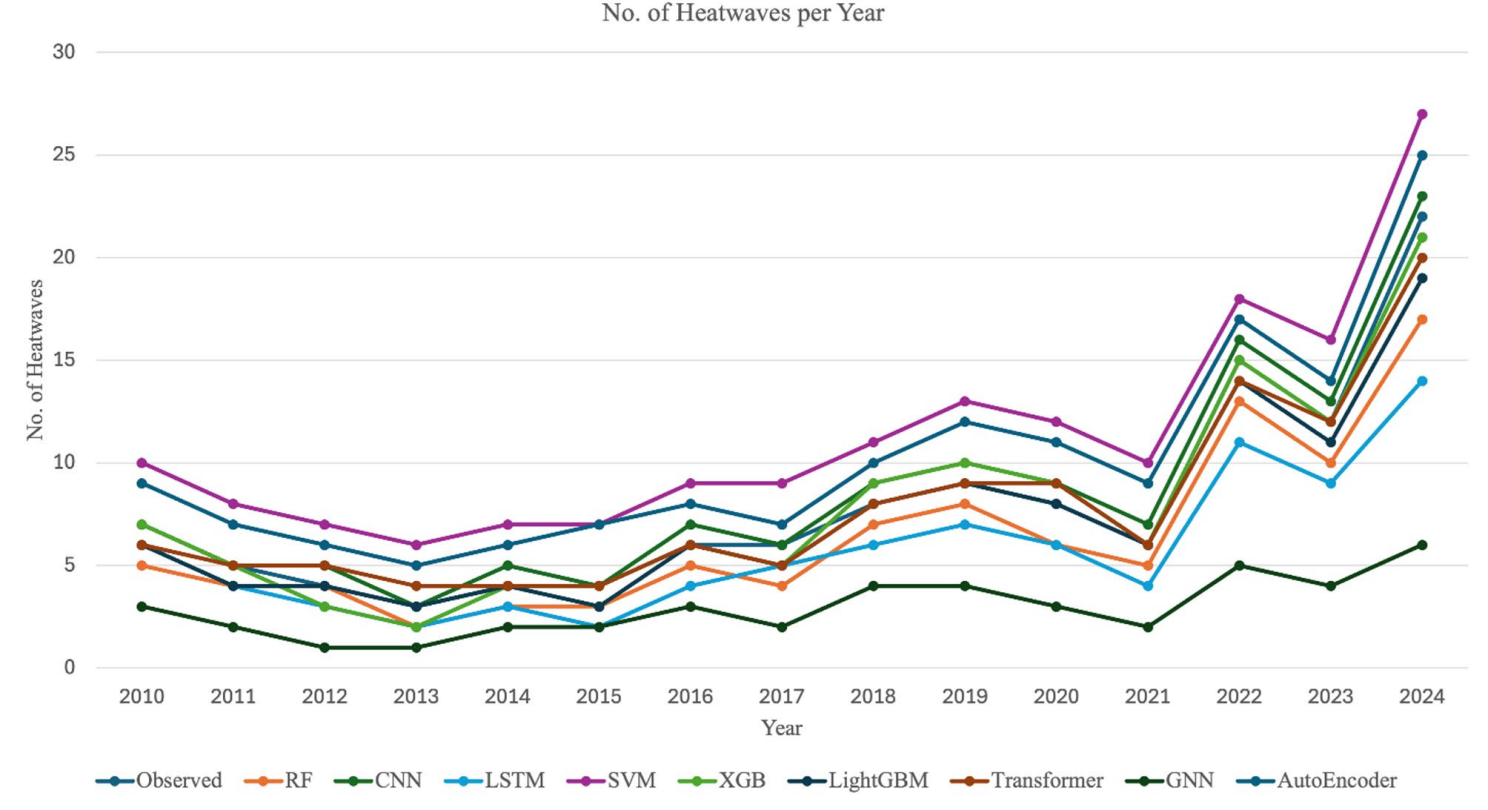
Predicted heatwave count for all nine models.

Figure 7 helps visualize the performance of various machine learning models across five key metrics: Precision, Recall, F1-Score, ROC AUC, and Accuracy. Each model is represented by a distinct line and shaded area, allowing for a comprehensive comparison of their performance profiles. The predicted values of the number of heatwaves for each year is shown in Fig. 8 for all the nine models considered in this research.

## Discussion

The Key Observations from the above results and from Fig. 5 are

- Model Performance Across Metrics:
  XGBoost, LightGBM, and Transformers exhibit strong performance across all metrics, with high values in Precision, Recall, F1-Score, ROC AUC, and Accuracy. Their shapes on the radar chart are larger and more evenly distributed, indicating consistent performance across the evaluated criteria.
  CNN also shows robust performance, particularly in Precision and Recall, though it slightly trails in F1-Score compared to the top performers.
- Areas of Weakness:
  SVM demonstrates high Recall but relatively low Precision, resulting in a shape that is stretched along the Recall axis. This reflects its ability to identify all heatwave events but with many false positives.
  LSTM has a notably poor performance in Precision, Recall, and F1-Score, evidenced by its small shape on the radar chart. Despite a reasonably high ROC AUC and Accuracy, its inability to predict heatwaves accurately is a significant drawback.
- Special Cases:
  GNN shows a limited performance with low Precision and Recall, as indicated by its small, less defined shape. This is likely due to the model’s unsuitability for the non-graph-structured time series data.
  Autoencoders are effective at identifying rare events (anomalies) but show limited classification capability, as illustrated by their high Recall but low Precision and F1-Score.

The major findings of this research work indicate that ensemble and boosting algorithms such as Random Forest, XGBoost, and LightGBM generally performed well in the heatwave prediction task, with high ROC AUC scores and balanced precision and recall metrics. These models’ ability to handle structured data and adjust for class imbalances contributed to their effective performance.

CNNs also showed strong results with highest classification accuracy, leveraging their pattern recognition capabilities to handle complex meteorological data. However, their requirement for large datasets and computational power can be a limitation in practical applications.

LSTM networks, designed for sequential data, struggled due to the extreme class imbalance. This highlights the challenge of using LSTMs for rare event prediction when the model’s focus on dominant patterns overshadows the minority class.

SVMs’ high recall but low precision underscores the trade-off between sensitivity and specificity, which is common in imbalanced datasets. Their performance suggests that while SVMs are effective at identifying positive instances, they may not be ideal for applications requiring high precision.

Transformers showed similar performance to gradient boosting methods, benefiting from their flexibility and ability to model complex dependencies. Despite their strong performance, their computational demands and complexity can be significant drawbacks.

GNNs and autoencoders for anomaly detection exhibited limitations. GNNs, not suited for non-graph-structured data, and autoencoders, better suited for anomaly detection rather than classification, underperformed in this context.

### Reasons for higher predicting skills of the chosen models

1. Ensemble learning and boosting models (XGBoost, LightGBM, Random Forest)

- *Strong Performance* Performance results show that these models consistently achieved high scores across all evaluation metrics.
- *Reason* XGBoost and LightGBM use gradient boosting techniques, hence they iteratively minimize errors and adjust weights, making them robust to noise and imbalanced data. Random Forest use multiple decision trees which reduces variance and improves generalization. Since these models handle structured tabular data effectively, they are well-suited for meteorological datasets.
- *Limitation*: May become computationally expensive when processing extremely large datasets.

2. Convolutional neural networks (CNNs)

- *Strong Performance* CNNs showed high accuracy (98.92%) and were among the best-performing models.
- *Reason* CNNs are seen to excel at detecting spatial patterns and non-linear relationships and are effective for learning complex dependencies in weather data. They have ability to extract high-level features automatically.
- *Limitation* Requires large training data. Computationally intensive, making real-time forecasting more challenging.

3. Transformer networks

- *Strong Performance* Transformers demonstrated performance comparable to XGBoost and LightGBM in terms of accuracy and recall.
- *Reason* Transformers process sequences in parallel enabling them to capture long-term dependencies efficiently. Their attention mechanism focuses on the most relevant data points for heatwave prediction.
- *Limitation* Computational cost is significantly higher than traditional machine learning models. Requires extensive hyperparameter tuning for optimal results.

4. Long short-term memory (LSTM) networks

- *Poor Performance* The model showed recall, precision, and F1-score of 0.00, making it ineffective for predicting heatwaves.
- *Reason* Usually LSTMs are designed for sequential data, but in this case, the extreme class imbalance made it difficult for the model to learn meaningful patterns for rare events. The network seems overfitted to the majority class (non-heatwave days), resulting in poor detection of heatwave events.
- *Improvement* Applying weighted loss functions or anomaly detection approaches could help improve LSTM’s performance.

5. Support vector machines (SVM)

- *Mixed Performance* SVM showed high recall (1.00) but extremely low precision (0.12), meaning it predicted nearly all events as heatwaves, leading to excessive false positives.
- *Reason* SVMs work well when the classes are separable, but in highly imbalanced datasets, they tend to favor the majority class or misclassify rare events.
- *Improvement* Adjusting the decision boundary or using weighted SVM will improve precision.

6. Graph neural networks (GNN)

- *Poor Performance* The GNN model failed to detect any heatwave events, achieving an F1-score of 0.00.
- *Reason* GNNs are designed for graph-structured data, where relationships between nodes are critical. However, weather data is not inherently graph-structured, making this model unsuitable.

7. Autoencoders for anomaly detection

- *Mixed Performance* Autoencoders showed high recall (0.94) but extremely low precision (0.07), leading to a large number of false positives.
- *Reason* Since heatwaves are rare events, autoencoders identified them as anomalies but could not distinguish between actual heatwaves and other temperature fluctuations.

While most of the existing literature have been carried out in different geographical locations and for different environmental settings, it becomes hard to compare our results with them. Still, CNN implementation by Jacques-Dumas et al.^5^ shows accuracy level of 95%. The Attention-Based Graph Neural Network implemented by Li, Peiyuan et al.^6^ provide an accuracy of 94.1%. Compared to these results, the accuracies obtained in this research work for the equatorial region scenario seems better at 98.92% for CNN.

To evaluate the effectiveness of the proposed machine learning models, a persistence model was implemented as a baseline. The persistence model operates under the assumption that tomorrow’s label is the same as today’s—a simplistic approach but useful for benchmarking.

The persistence model achieved a high overall accuracy of 96.47%, primarily due to the dominance of the majority class (non-heatwave days). However, it failed to identify any heatwave events, resulting in a recall of 0.00 and an ROC AUC of 0.49, indicating no discriminative ability for rare events. The confusion matrix revealed that while it correctly predicted 985 non-heatwave days, it misclassified all 18 actual heatwave instances.

These results highlight a key limitation of relying on overall accuracy in imbalanced classification problems. Despite the high accuracy, the persistence model performs no better than random guessing in detecting heatwaves. In contrast, machine learning models such as CNN, XGBoost, and LightGBM achieved much higher ROC AUC values (> 0.99) and balanced precision-recall scores, demonstrating their ability to detect minority class events effectively. This underscores the necessity of using advanced modeling techniques and appropriate evaluation metrics when addressing rare event prediction in climatological datasets.

Persistence Model Evaluation:

Accuracy: 0.9647404505386875.

ROC AUC: 0.49102691924227315

Confusion Matrix: $$\left[\begin{array}{cc}985& 18\\ 18& 0\end{array}\right]$$

**Table:** 

Classification report of persistence prediction model				
	Precision	Recall	f1-score	Support
0	0.98	0.98	0.98	1003
1	0	0	0	18
Accuracy			0.96	1021
Macro average	0.49	0.49	0.49	1021
Weighted average	0.96	0.96	0.96	1021

The current models predict heatwave events for the following day. The main significance of this research lies in the level of accuracy in prediction in spite of the possibility of similar prevalence in consecutive days. As discussed in^52–55^ the prediction accuracy and heatwave projection greatly depend on the persistence of events in terms of temporal dependencies of the time series data. Earlier methods described in^22–25^ disrupt the temporal dependencies, making them unsuitable for predicting events based on sequential data. To address these challenges, this study explores alternative machine learning techniques and class weighting techniques for handling class imbalance in the temporal data. This leads to improved heatwave detection accuracy without compromising temporal dependencies leading to high levels of persistence prediction accuracy (98.92% for CNN, 98.82% for Extreme Gradient Boosting, 98.63% for Light GBM, 98.12% for Random Forest, 98.06% for LSTM and Transformer Networks).

Extending predictions beyond one day can be achieved by recursively feeding model outputs as inputs for future predictions. However, this approach results in a multiplicative decrease in confidence due to accumulated prediction errors. While trends in the number and duration of heatwaves can be forecasted using historical data and climate models, long-term predictions are inherently uncertain due to the complexity of climate dynamics, external influences, and feedback mechanisms. Predicted values can be incorporated into Figs. 2 and 3 based on model projections. However, as mentioned earlier, the warmest months are May is challenging and can lead to significant deviations from actual values. The persistence model, which assumes that tomorrow’s weather condition (heatwave or not) will be the same as today’s, was implemented as a baseline to evaluate the added value of the machine learning models. While it achieved a seemingly high overall accuracy of 96.47%, this performance was heavily biased by the overwhelming number of non-heatwave days in the dataset. Critically, it failed to identify a single heatwave event, resulting in a recall of 0.00 and an ROC AUC of just 0.49, indicating no real predictive skill for the minority class. In contrast, the evaluated machine learning models—particularly CNN, XGBoost, and LightGBM—achieved significantly higher ROC AUC scores (greater than 0.99) and demonstrated balanced performance across all key metrics, including precision, recall, and F1-score. These results clearly show that, although the persistence model may appear accurate due to class imbalance, it lacks the ability to detect heatwaves altogether. Therefore, the use of more complex models is not only justified but essential for capturing the rare and impactful events that simplistic baselines entirely overlook.

### Novel findings of this research

- First Large-Scale Machine Learning Benchmark for Heatwave Prediction in an Equatorial Region: While most heatwave studies focus on temperate climates, this research is among the first to systematically evaluate multiple machine learning models for heatwave forecasting in an equatorial setting (Tamil Nadu, India). The high humidity and unique monsoonal influences in this region affect heatwave dynamics differently from those in drier climates, making these findings regionally significant and globally informative.
- Comparative Analysis of Nine Machine Learning Models: This study provides one of the most comprehensive evaluations of traditional, ensemble, and deep learning models for heatwave prediction. CNNs and ensemble models (XGBoost, LightGBM) outperformed LSTMs and Transformers in short-term forecasting, challenging the assumption that sequential models (LSTMs) are best suited for time-series weather predictions.
- Demonstrating the Impact of Class Imbalance and the Limitations of SMOTE in Time-Series Forecasting: Unlike most studies that apply Synthetic Minority Over-Sampling Technique (SMOTE) to balance classes, this research demonstrates that SMOTE disrupts temporal dependencies in heatwave prediction. Instead, class weighting and anomaly detection were suggested as better alternatives, providing a new strategy for handling rare event prediction in time-series data.

New Insights into the Failure of Graph Neural Networks (GNNs) for Heatwave Prediction: This study is one of the first to apply Graph Neural Networks (GNNs) to meteorological data for heatwave classification. Unlike other studies where GNNs excel in structured spatial data, they performed poorly in heatwave prediction, indicating that weather data lacks an inherent graph structure necessary for GNN effectiveness.

### Comparison with other researches

While most existing studies have been conducted in different geographical locations and environmental settings, making direct comparisons challenging, some relevant works provide a useful benchmark for evaluating the significance of our results. For instance, the CNN implementation by Jacques-Dumas et al.^5^ achieved an accuracy of 95%, while the Attention-Based Graph Neural Network developed by Li, Peiyuan et al.^6^ reported an accuracy of 94.1%. These models demonstrated strong predictive capabilities; however, it is important to note that they were trained and validated in different climatic regions, which may have distinct meteorological conditions and data distributions. As a result, their applicability to equatorial regions, which experience unique temperature variations and atmospheric patterns, remains uncertain.

**Fig. 9:**
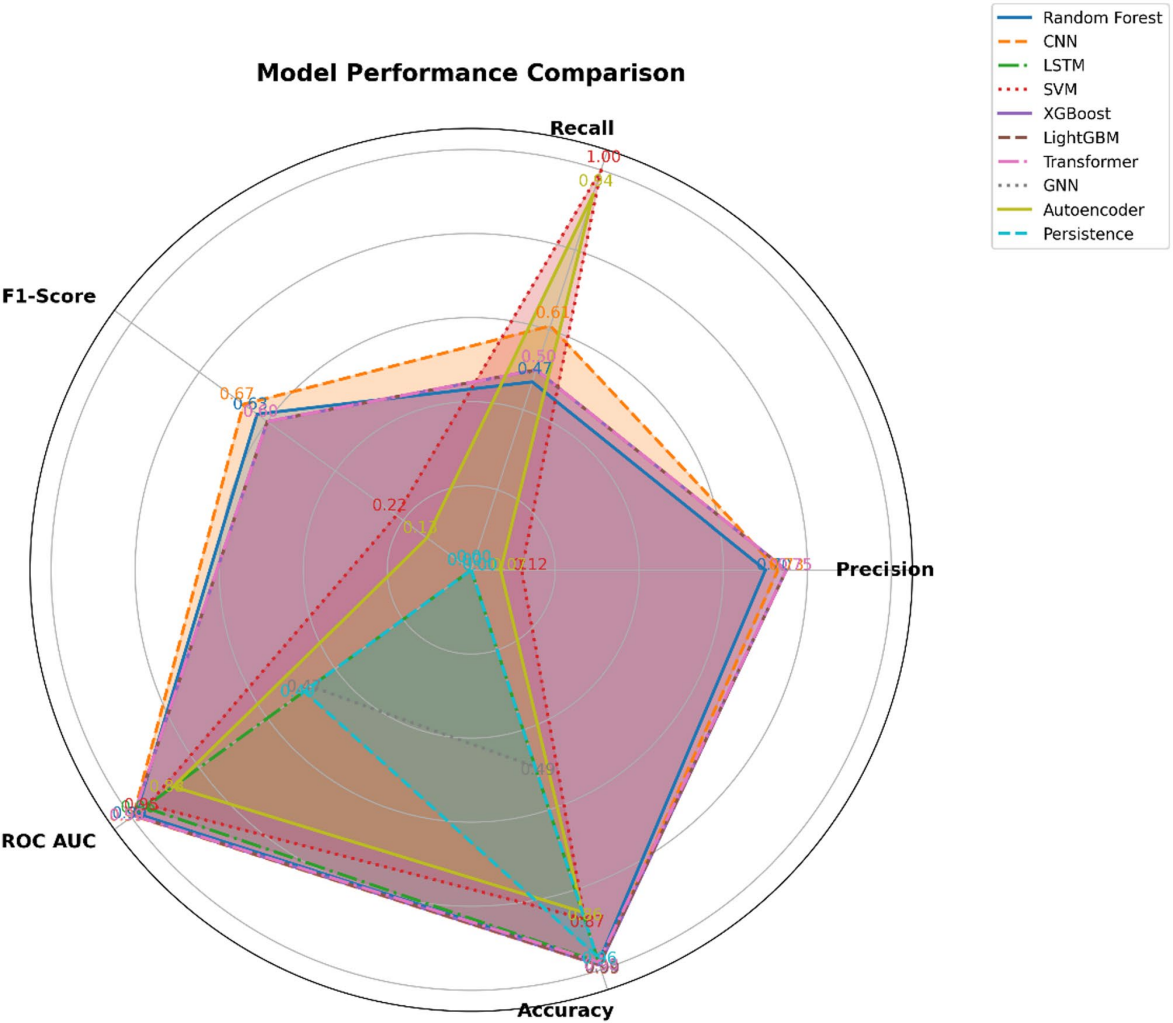
Radar chart for overall performance along with persistence prediction model.

In contrast, our research is specifically tailored to the equatorial region, where extreme heat events exhibit different characteristics compared to temperate and arid zones. Heatwave prediction in this region presents additional challenges due to factors such as high humidity levels, seasonal monsoons, and fluctuating cloud cover, all of which can influence temperature trends and make forecasting more complex. Despite these difficulties, our CNN model achieved a significantly higher accuracy of 98.92%. Figure 9 shows the comparison of the implemented model with the persistence prediction model.

These factors make direct benchmarking with prior studies difficult, as existing research often focuses on different climatic zones, datasets, and methodologies.

## Conclusion

This study presents a benchmarking of machine learning models for heatwave prediction in an equatorial region, addressing critical challenges in forecasting accuracy, model selection, and class imbalance handling. The results show that CNNs and ensemble methods (XGBoost, LightGBM) outperform LSTMs and Transformers, in contrast to the conventional assumptions about sequential models in climate forecasting. A key contribution is the finding that SMOTE-based oversampling disrupts time-series continuity, and class weighting is a more effective strategy for handling heatwave rarity. Additionally, the study highlights the ineffectiveness of Graph Neural Networks (GNNs) for heatwave prediction, refining their role in climate modeling. By directly comparing predicted vs. observed heatwave events, this research enhances the practical applicability of machine learning in extreme weather forecasting. Future work should explore hybrid models combining CNNs with boosting techniques and develop longer lead-time predictions while maintaining confidence levels.

The evaluation demonstrates that while various models offer different strengths, ensemble methods like Random Forest, XGBoost, and LightGBM, as well as CNNs and Transformers, performed notably well in heatwave prediction. Their ability to handle imbalanced data and capture complex patterns proved beneficial. Conversely, LSTM networks and SVMs, despite their potential, faced challenges related to class imbalance and computational efficiency. GNNs and autoencoders were less effective due to misalignment with the data’s structure and task requirements.

The results of this study underscore the effectiveness of ensemble and boosting algorithms for heatwave prediction, suggesting that future research should focus on optimizing these models for better handling of class imbalance. Incorporating advanced resampling techniques or innovative approaches to address class imbalance in time series data could further enhance model performance.

Unlike many prior works that focus on temperate or continental regions, this research specifically addresses the unique climatic challenges of Tamil Nadu, India, where high humidity and monsoonal patterns distinctly influence heatwave dynamics. A comprehensive benchmark of nine diverse models—including ensemble methods, deep learning architectures, and anomaly detection techniques—which not only exposes the limitations of commonly used approaches like SMOTE for time-series data but also highlights the superior performance of CNNs and gradient boosting methods in predicting rare events have been presented. Additionally, by directly comparing model predictions against observed heatwave events, this study provides actionable insights that strengthen the practical applicability of these models for climate adaptation strategies.

Additionally, exploring hybrid models that are capable of combining the strengths of various algorithms, such as integrating CNNs with ensemble methods, may offer a promising avenue for improving prediction accuracy. Future work should also consider the scalability and real-time applicability of these models to ensure their effectiveness in practical scenarios.

Moreover, there is a need to expand the dataset to include more diverse geographical regions and climatic conditions. This would help in validating the robustness of the models and their generalizability to different contexts.

Accurate and early prediction of heatwaves is necessary for mitigating their adverse impacts on agriculture, human health, animal health and infrastructure. The findings of this study provide a valuable foundation for developing more effective predictive models. By leveraging the strengths of ensemble methods and addressing the limitations of existing approaches, future research can contribute to better preparedness and resilience against the increased threat of heatwaves in the context of climate change.

## Supplementary Information

Below is the link to the electronic supplementary material.

Supplementary Information.

## Data Availability

Data will be made available upon request from the corresponding author Anita Christaline Johnvictor.
