# Supplementary material for: Comparative analysis of machine learning approaches for heatwave event prediction in India
Source: Sci Rep. 2025 Jul 1;15:22431. doi: 10.1038/s41598-025-04634-9 (PMC12216918; doi:10.1038/s41598-025-04634-9)
Supplement: Supplementary file 1 — Supplementary Information. [file 41598_2025_4634_MOESM1_ESM.docx]

**Comparative Analysis of Machine Learning Approaches for Heatwave Event Prediction in India**

| Ritesh Choudhary V^1^  School of Computer Science  and Engineering,  Vellore Institute of  Technology, Chennai campus, Chennai,  India.  [riteshchoudhary.v2022@vitstudent.ac.in](mailto:riteshchoudhary.v2022@vitstudent.ac.in) | Anita Christaline Johnvictor.^2^*  Centre for Neuro Informatics,  Vellore Institute of Technology, Chennai campus, Chennai, India.  [anitachristaline.j@vit.ac.in](mailto:anitachristaline.j@vit.ac.in) | N. Prem Sankar^3^  Centre for Healthcare Advancement, Innovation and Research, Vellore Institute of Technology, Chennai campus, Chennai, India.  [premsankar.n@vit.ac.in](mailto:premsankar.n@vit.ac.in) |
| --- | --- | --- |

**Supplementary Information**

This section presents the results obtained for each type of model implemented in this research work.


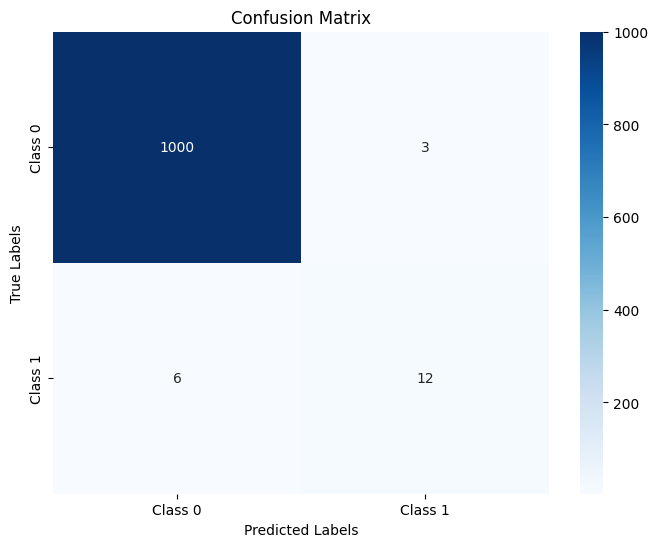


Supplementary Figure 1. Confusion matrix for Random Forest


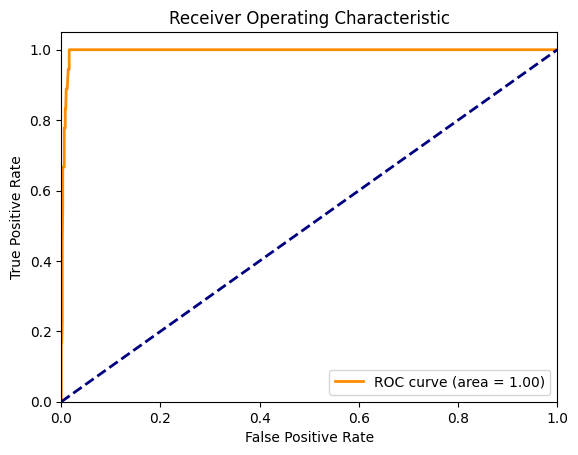


Supplementary Figure 2. ROC curve for Random Forest


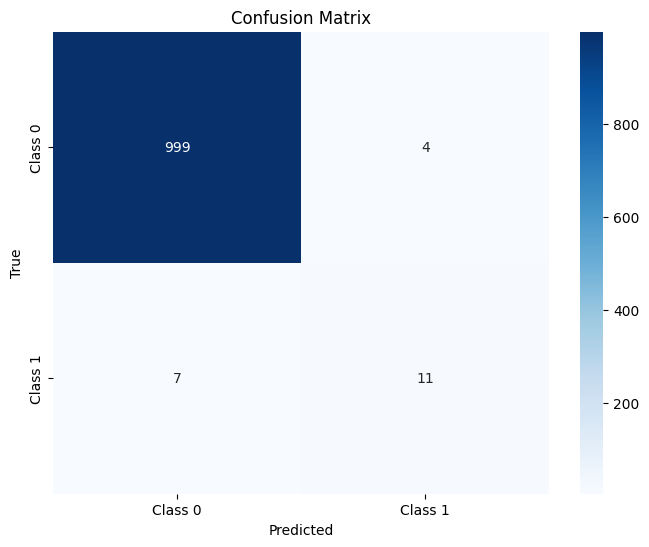


Supplementary Figure 3. Confusion matrix for CNN


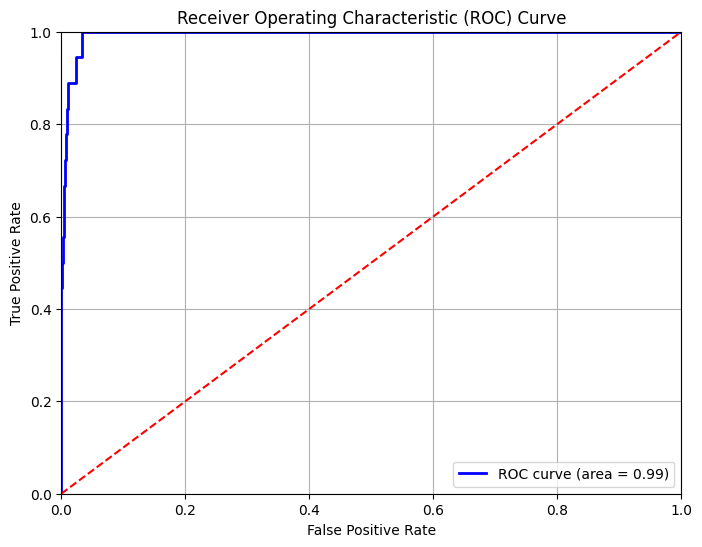


Supplementary Figure 4. ROC curve for CNN


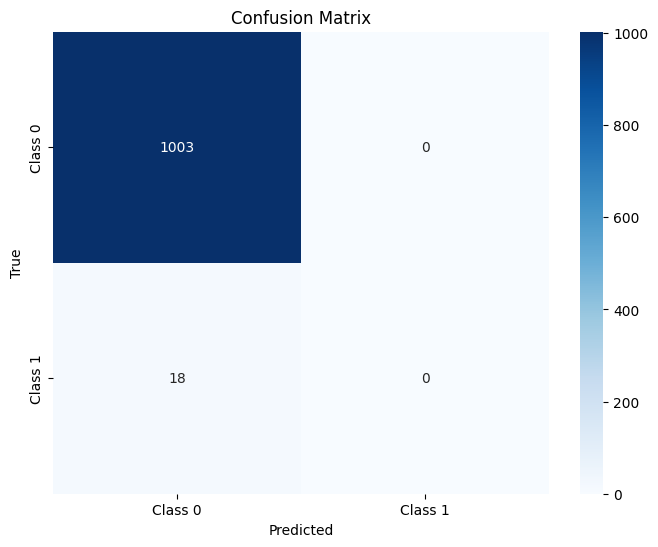


Supplementary Figure 5. Confusion matrix for LSTM


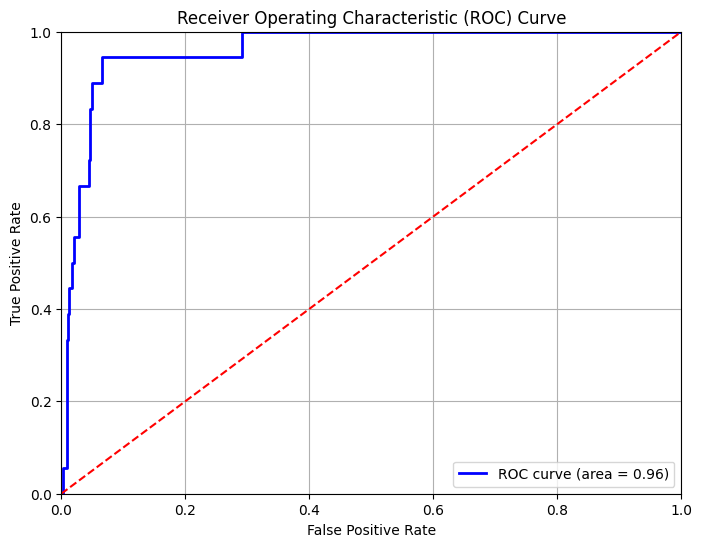


Supplementary Figure 6. ROC curve for LSTM


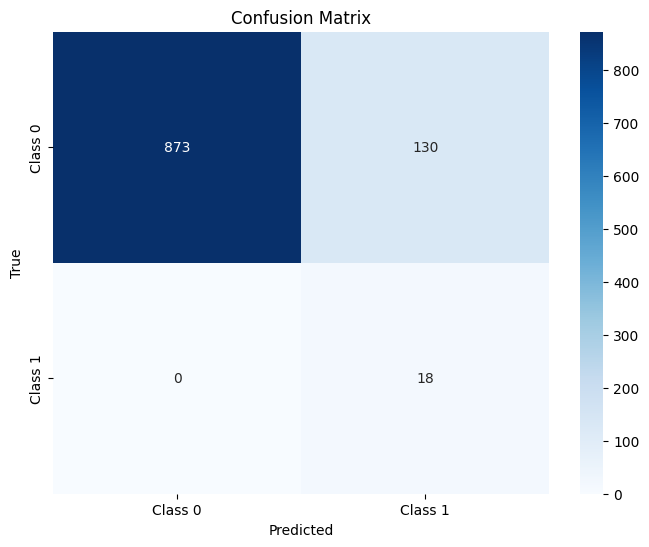


Supplementary Figure 7. Confusion matrix for SVM


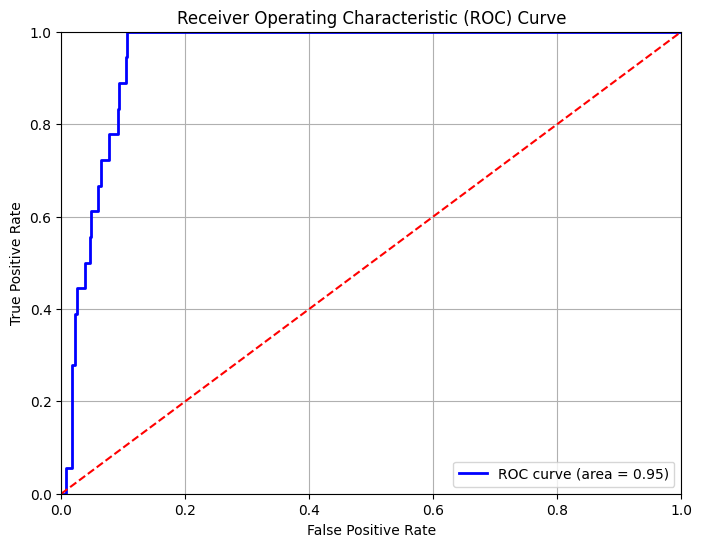


Supplementary Figure 8. ROC curve for SVM


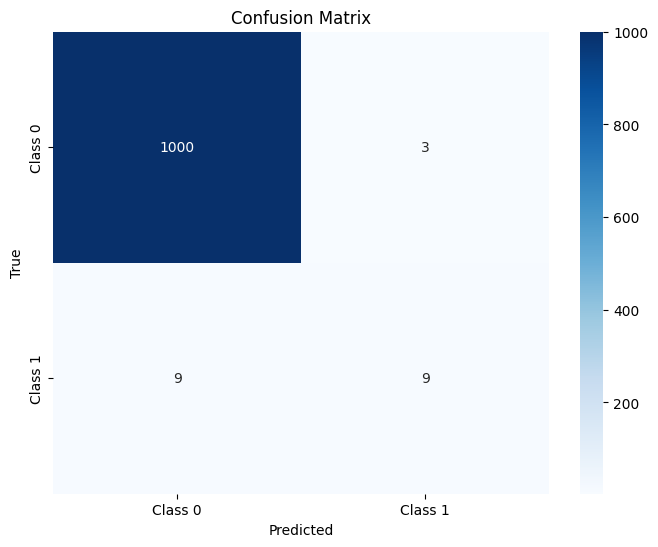


Supplementary Figure 9. Confusion matrix for XGBoost


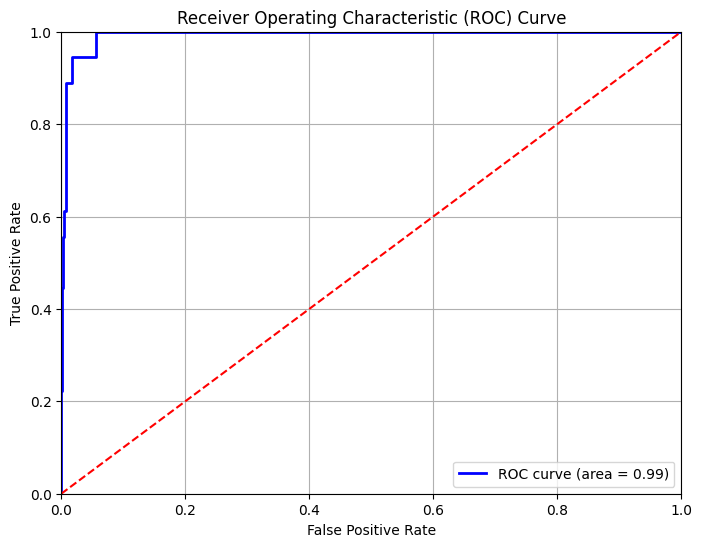


Supplementary Figure 10. ROC curve for XGBoost


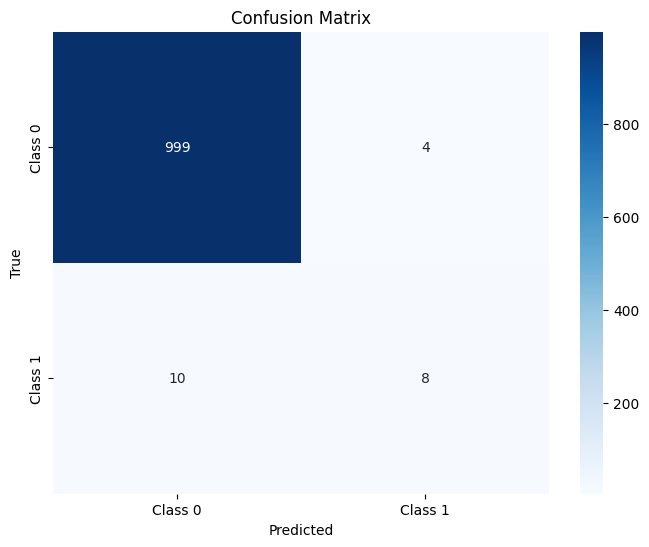


Supplementary Figure 11. Confusion matrix for LightGBM


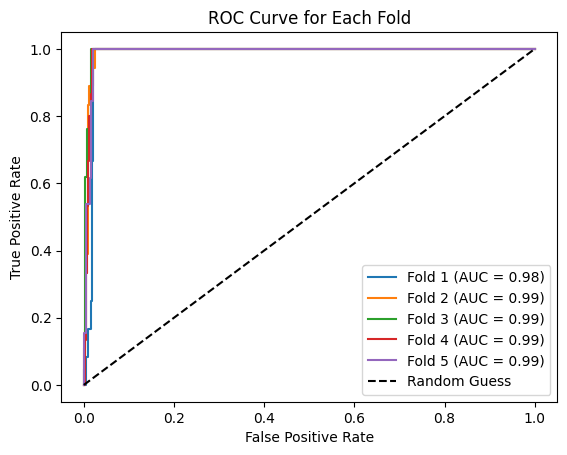


Supplementary Figure 12. ROC curve for LightGBM


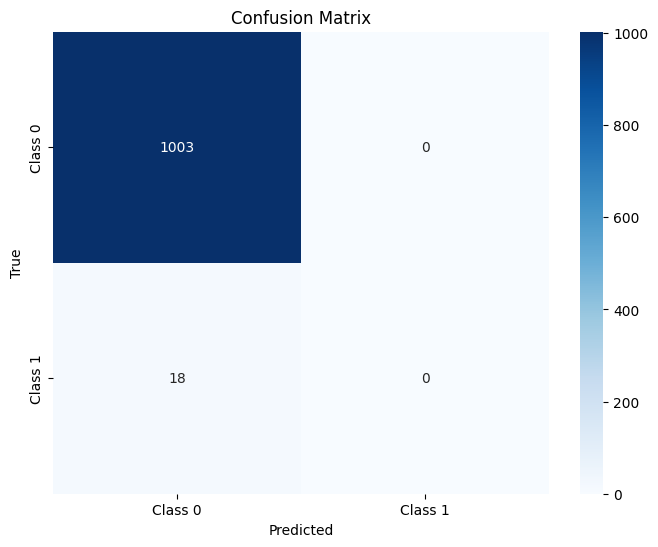


Supplementary Figure 13. Confusion matrix for Transformer Networks


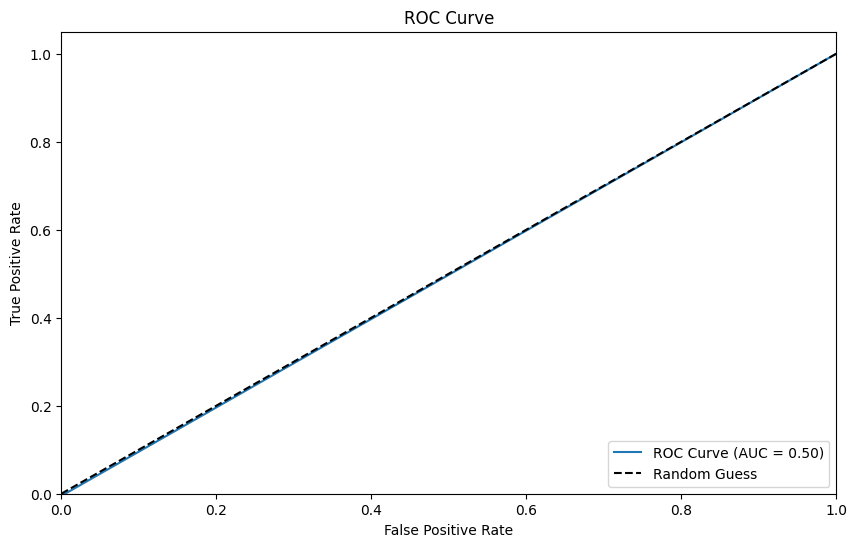


Supplementary Figure 14. ROC curve for Transformer Networks


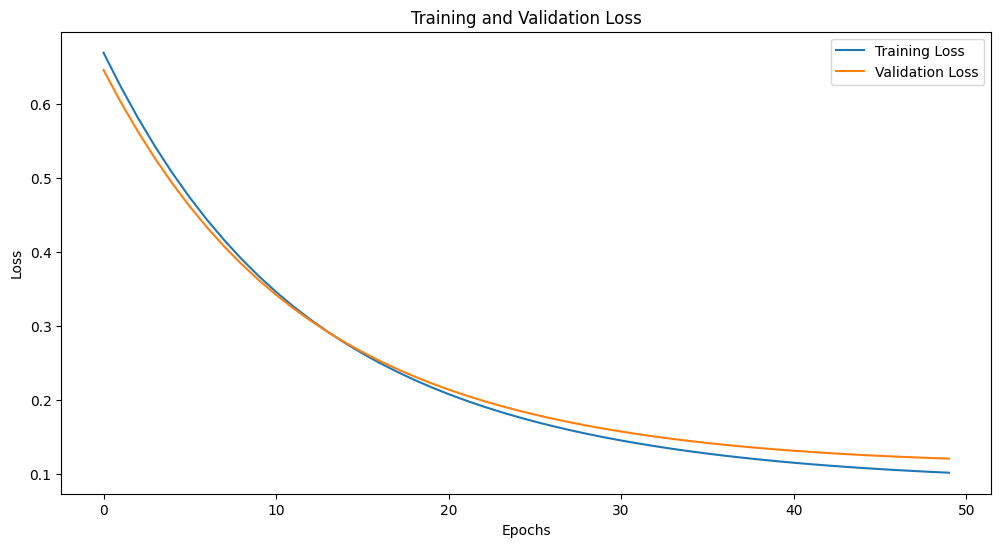


Supplementary Figure 15. Training and Validation Loss in Transformer Networks


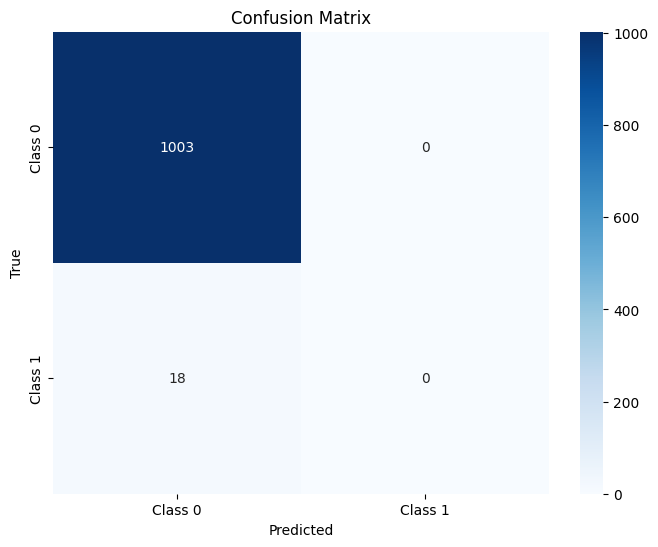


Supplementary Figure 16. Confusion matrix for GNN


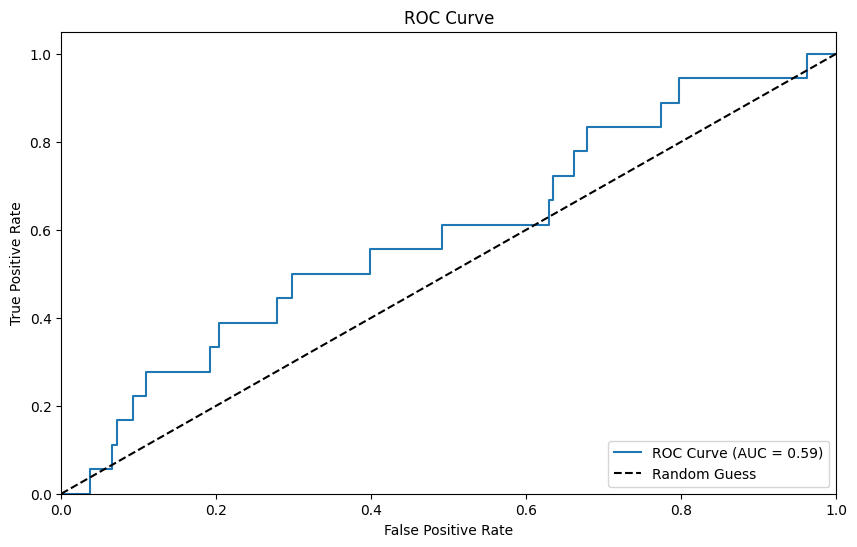


Supplementary Figure 17. ROC curve for GNN


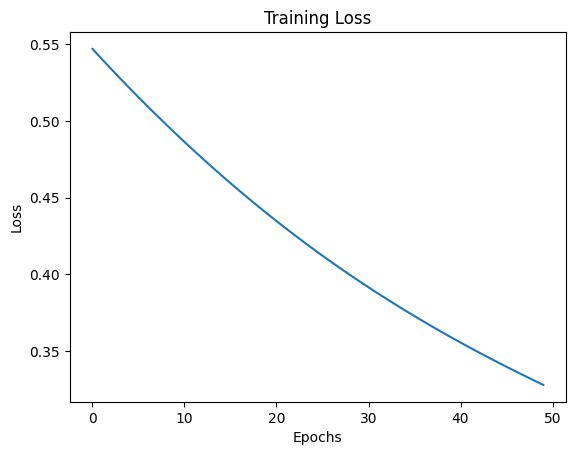


Supplementary Figure 18. Training Loss in GNN.


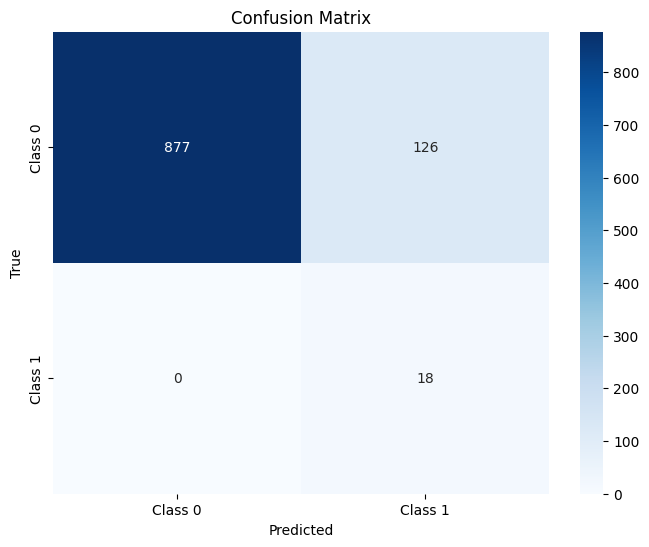


Supplementary Figure 19. Confusion matrix for Autoencoders


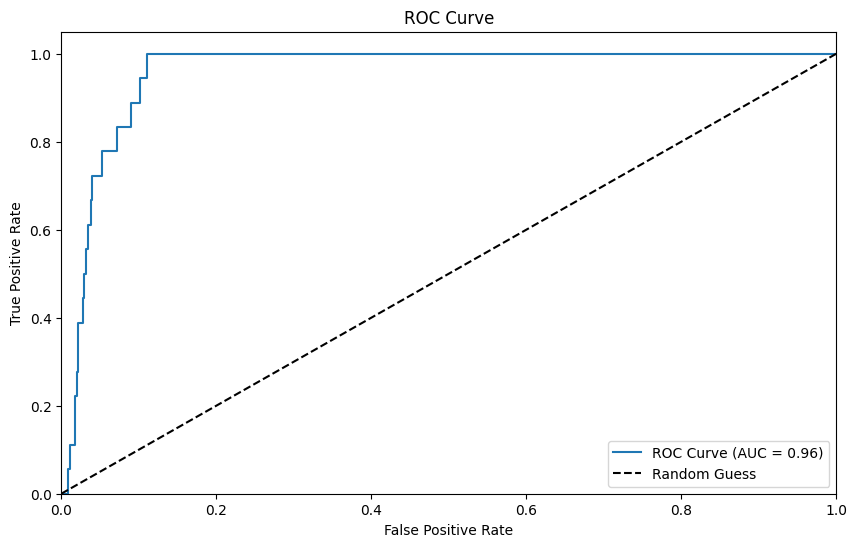


Supplementary Figure 20. ROC curve for Autoencoders


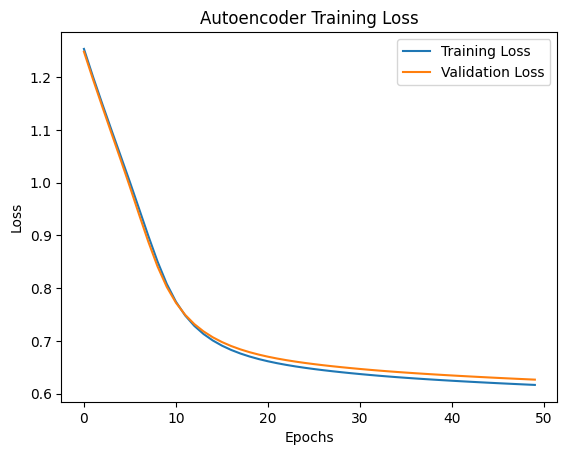


Supplementary Figure 21. Training Loss in Autoencoders

[Monthly climate in Chennai, India](https://nomadseason.com/climate/india/tamil-nadu/chennai.html#:~:text=The%20warmest%20months%20in%20Chennai%20are%20May%2C%20June,%C2%B0C%20%2878%20-%2079%20%C2%B0F%29%20throughout%20the%20day.)


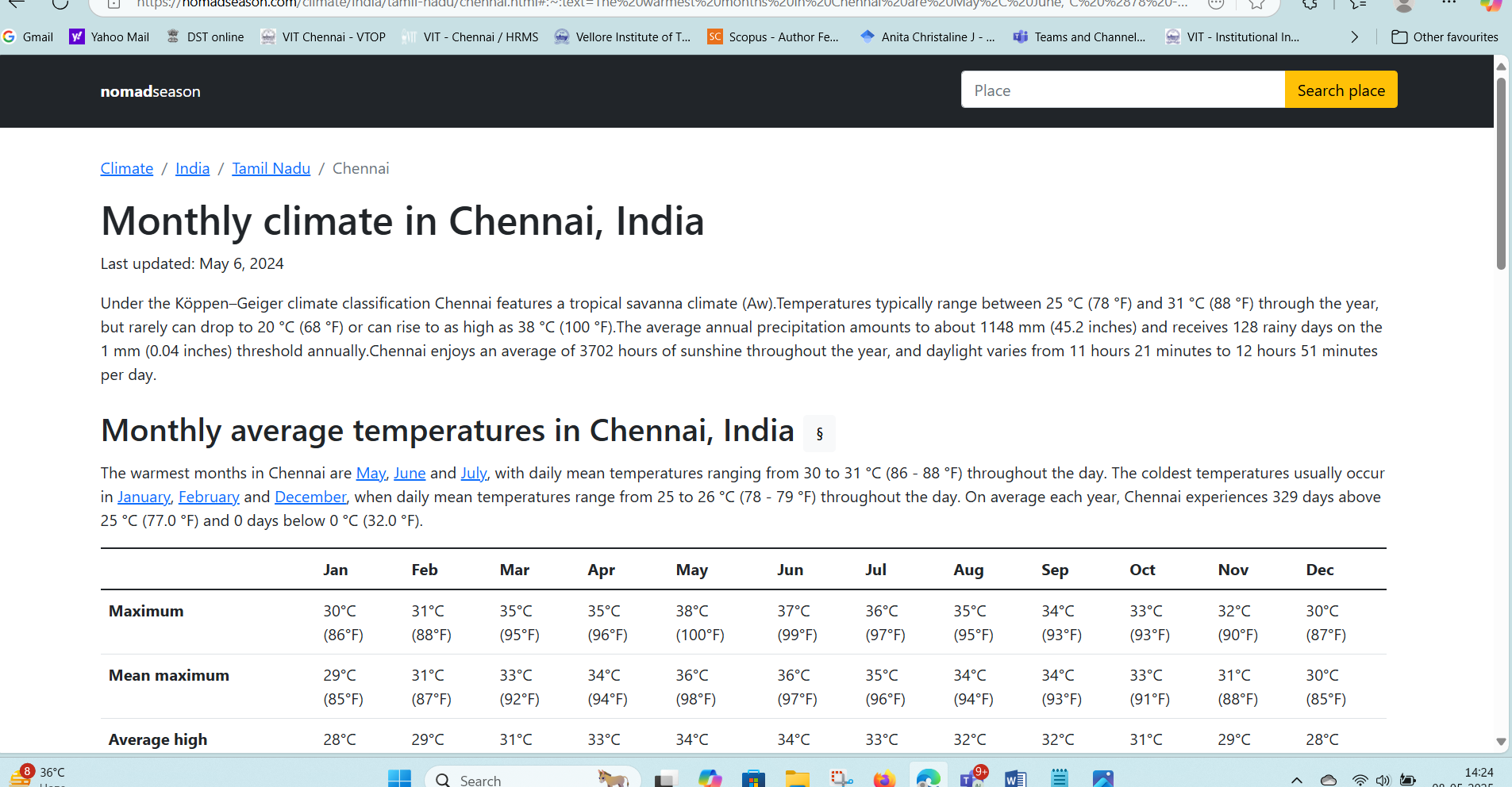


Supplementary Figure 22. Monthly Climate in Chennai.

[Weather Data Documentation | Visual Crossing](https://www.visualcrossing.com/resources/documentation/weather-data/weather-data-documentation/)


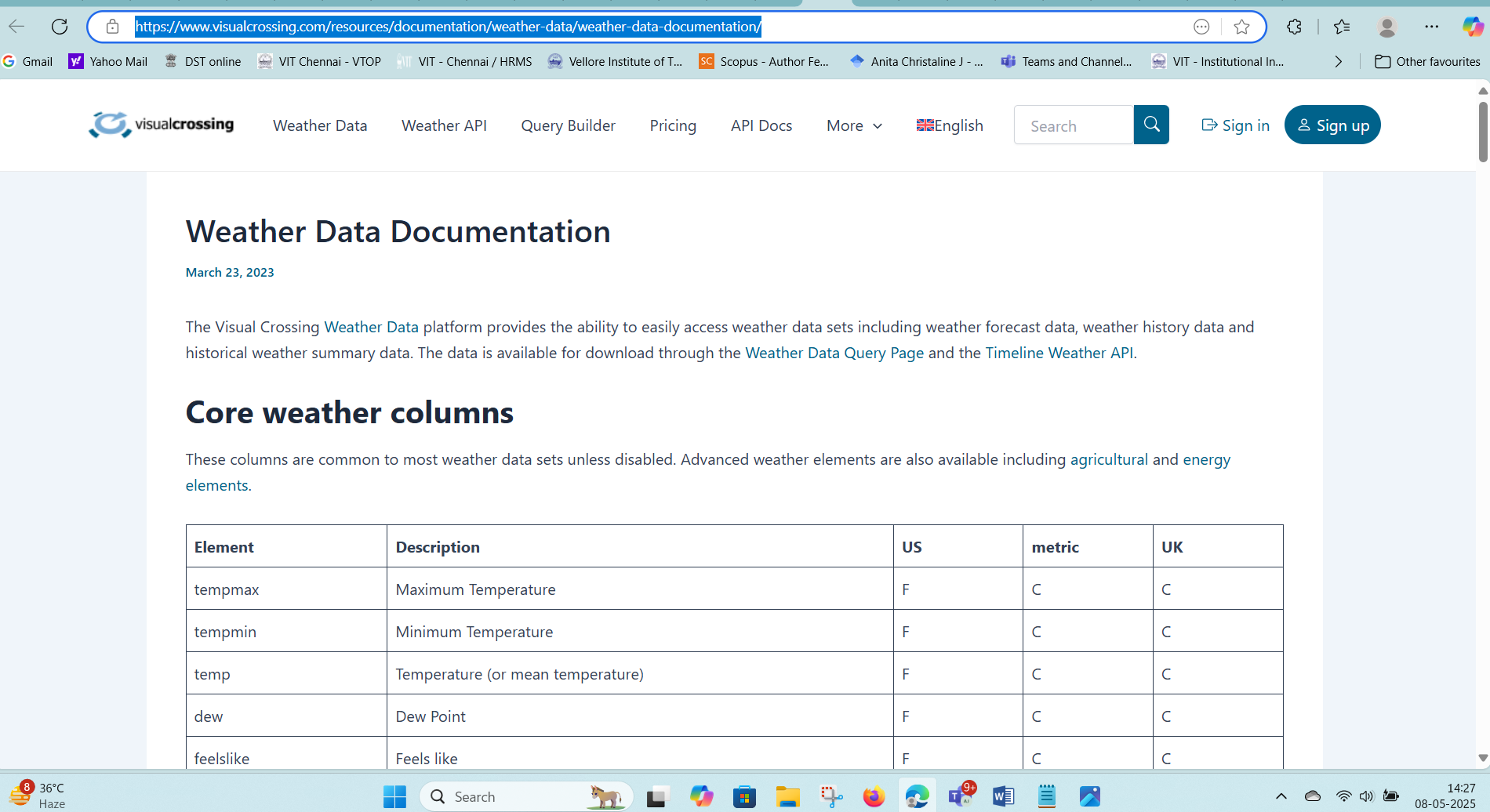


Supplementary Figure 23. Weather data from the dataset used in this research.
